# Supplementary material for: Evaluation of the Antiviral Activity of Tabamide A and Its Structural Derivatives against Influenza Virus
Source: Int J Mol Sci. 2023 Dec 9;24(24):17296. doi: 10.3390/ijms242417296 (PMC10744247; doi:10.3390/ijms242417296)
Supplement: Supplementary file 1 [file ijms-24-17296-s001.zip › ijms-2767565-supplementary.pdf]

Electronic Supplementary Information

Evaluation of the Antiviral Activity of Tabamide A and Its Structural Derivatives against Influenza Virus

Soo Yong Shin [1], Joo Hee Lee [1], Jin Woo Kim [1], Wonkyun Ronny Im [1], Kongara Damodar [2,†], Hyung Ryeol Woo [2], Won-Keun Kim [3], Jeong Tae Lee [2,*] and Sung Ho Jeon [1,*]

1 Department of Life Science and Multidisciplinary Genome Institute, Hallym University, Chuncheon 24252, Republic of Korea

2 Department of Chemistry and Institute of Applied Chemistry, Hallym University, Chuncheon 24252, Republic of Korea

3 Department of Microbiology and Institute of Medical Science, College of Medicine, Hallym University, Chuncheon 24252, Republic of Korea

* Correspondence: jtshl@hallym.ac.kr (J.T.L.); sjeon@hallym.ac.kr (S.H.J.)

† Present address: East Coast Life Sciences Institute, Gangneung-Wonju National University, Gangneung, 25457, Republic of Korea.

All chemicals were obtained from commercial suppliers and were used without further purification unless stated otherwise. All solvents used for reactions were freshly distilled from proper dehydrating agents under argon gas. All solvents used for chromatography were purchased and directly used without further purification. ^{1}H-NMR spectra were recorded on a JNM-ECZRS (400 MHz) FT-NMR and 100 MHz (JEOL Ltd., Tokyo, Japan) for ^{13}C, with the chemical shift (δ) reported in parts per million (ppm) downfield relative to TMS and the coupling constants (*J*) quoted in Hz. Peak splitting patterns were abbreviated as s (singlet), d (doublet), t (triplet), q (quartet), dd (doublet of doublet) and m (multiplet) and $CDCl_3$/acetone-d_6/DMSO-d_6 was used as a solvent. Mass spectra were recorded on JMS-700 (JEOL Ltd., Tokyo, Japan) spectrometer. Melting points were measured on a MEL-TEMP II (Triad Scientific, Manasquan, NJ, USA) apparatus using open capillary tubes and were uncorrected.

Reactions were monitored by thin-layer chromatography (TLC) on silica gel 60 F_{254} (Merck, Darmstadt, Germany) and spots were visualized under UV light or by staining with *p*-anisaldehyde and phosphomolybdic acid (PMA) stain. Chromatographic purification was carried out using silica gel 60 [230−400 mesh (40−63 µm), Merck, Darmstadt, Germany].

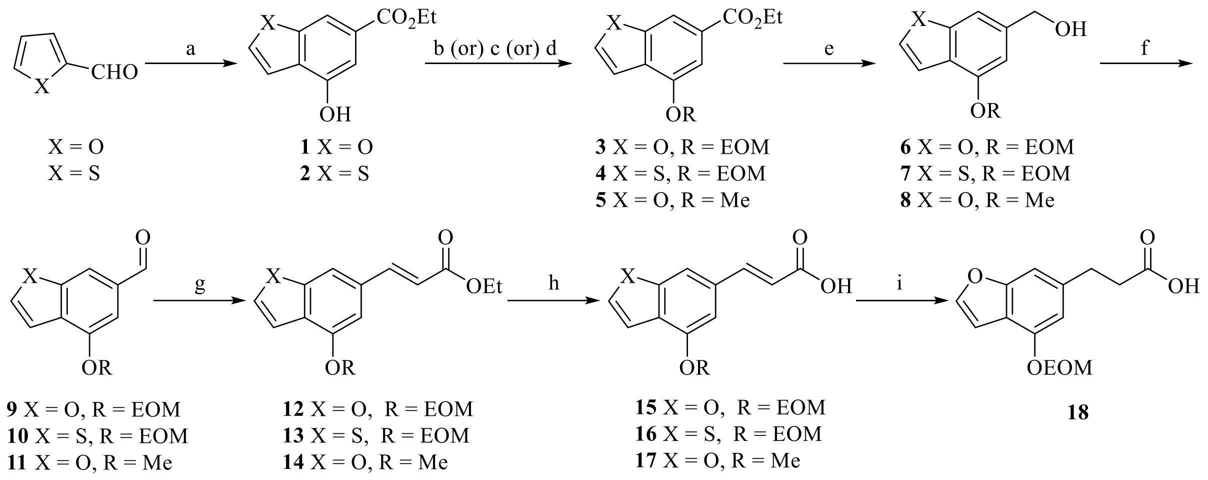

Scheme S1. Reagents and conditions: (a) (i) Ethylsuccinate, sodium ethoxide, reflux, 4 h; (ii) AcONa, Ac_2O, 155 ºC, 8 h; (iii) K_2CO_3, MeOH, reflux, 1 h, 43.6% (**1**), 42.8 (**2**) (over 3 steps); (b) chloromethyl ethyl ether (EOM-Cl), tetrabutylammonium iodide, K_2CO_3, acetone, 0 ºC - rt, 15 h, 87.0% (**3**); (c) EOM-Cl, NaH (60% in mineral oil), DMF, 0 ºC-rt, 7 h, 87.9% (**4**); (d) MeI, K_2CO_3, DMF, rt, 4 h, 95.0% (**5**); (e) $LiAlH_4$, THF, 0 ºC-rt, 2–3 h, 98.0% (**6**), 96.7% (**7**), 97.0% (**8**); (f) IBX, DMSO, rt, 2–3 h, 95.4% (**9**), 95.0% (**10**), 94.0% (**11**); (g) triethyl phosphonoacetate, *ᵗ*BuOK, DMF, 0 ºC, 30 min then rt, 4–5 h, 90.0% (**12**), 94.5% (**13**), 86.2% (**14**); (h) KOH, EtOH, reflux , 3–4 h, 92.0% (**15**), 70.5% (**16**), 50.8% (**17**); (i) H_2, Pd/C (10%), EtOAc, rt, 3–4 h, 91.6%.

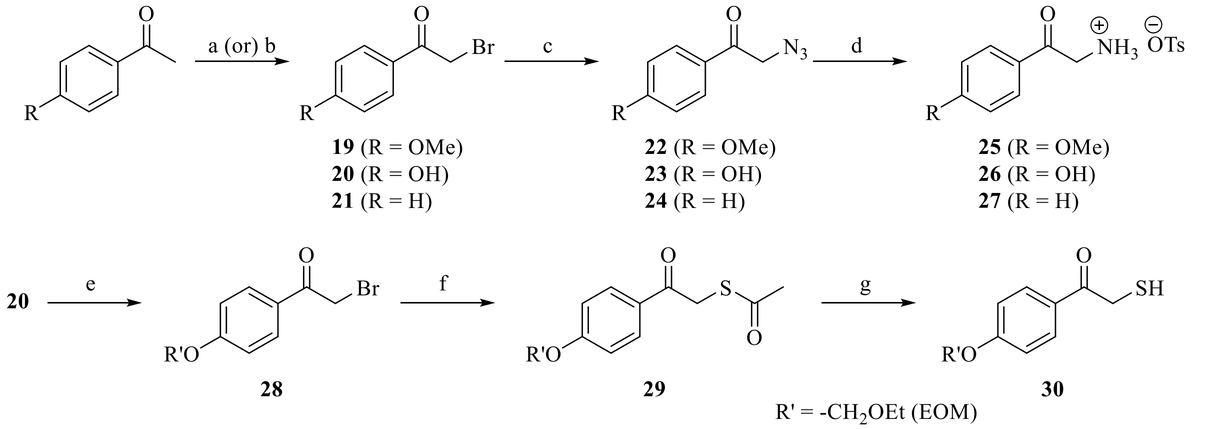

Scheme S2. Reagents and conditions: (a) NBS/*p*TSA.H_2O, $CHCl_3$, rt, 27 h, 78.7% (**19** R = OMe); (b) $CuBr_2$, $CHCl_3$/EtOH reflux, 3.5 h, 69.6% (**20** R = OH); (c) NaN_3, MeOH/H_2O, 0 ºC - 2h then 15–20 ºC, 2.5–3.5 h, 94.0% (**22**), 98.0% (**23**), 96.9% (**24**); (d) PPh_3/*p*TSA.H_2O, THF, rt, 20–24 h, 72.0% (**25**), 36.0% (**26**), 54.0%

(**27**); (e) EOM-Cl, DIPEA, CH_2Cl_2, 0 ºC – rt, 2 h, 77.2%; (f) potassium thioacetate, THF, rt, 4 h, 96.5%; (g) NaOH, MeOH, rt, 1 h, 82.6%.

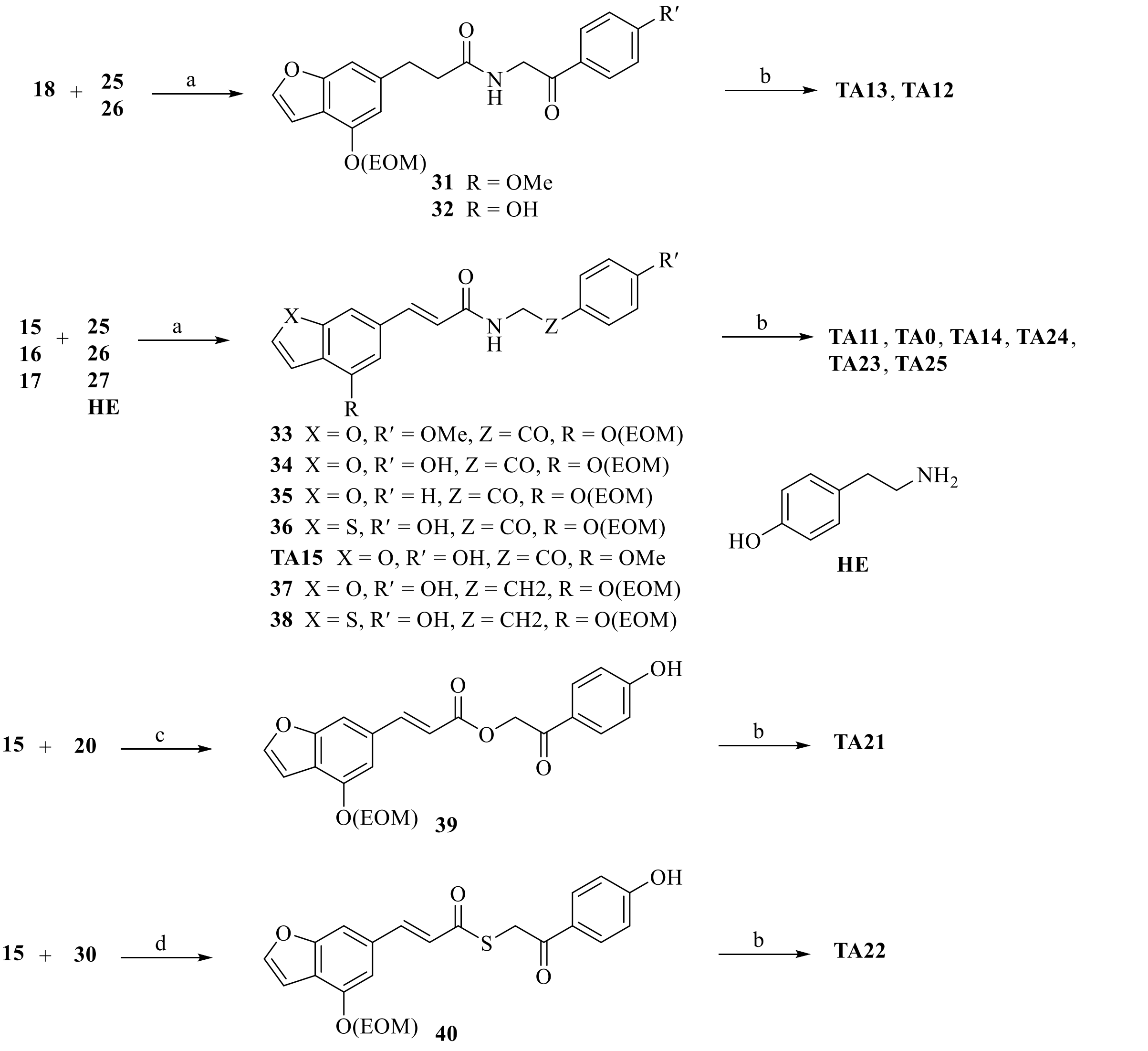

Scheme S3. Reagents and conditions: (a) HATU/NMM, DMF, 15–20 ºC-rt, 4.5–9 h, 28.3–96.0%; (b) 1N HCl aq., MeOH, 55–60 ºC 1 h, 63.0–94.5%; (c) K_2CO_3, DMF, rt, 3–15 h, 73.2% (d) EDCI/DMAP, CH_2Cl_2, rt, 2 h, 71.4%.

Ethyl 4-hydroxybenzofuran-6-carboxylate (1):

To a stirred solution of furfural (1.29 mL, 15.611 mmol) and diethyl succinate (3.24 mL, 19.474 mmol, 1.25 equiv.) in anhydrous EtOH (24 mL) was added sodium ethoxide (2.34 g, 34.392 mmol, 2.2 equiv.) under nitrogen atmosphere at room temperature. The resulting mixture was refluxed for 4 h. After completion of the reaction, cool to room temperature and

the solvent was removed under reduced pressure. The crude pH was adjusted to 1–3 with 3N HCl and extracted with EtOAc (2 x 75 mL). The combined organic layer was washed with 10% aqueous Na_2CO_3 (2 x 55 mL). The combined aqueous layer was acidified to pH 1–3 with 3N HCl and conc. HCl and extracted with EtOAc (2 x 90 mL). The combined organic layer was washed with H_2O (2 x 45 mL) and brine (40 mL), dried over anhydrous sodium sulfate and concentrated *in vacuo*. The crude product and AcONa (1.39 g, 16.944 mmol, 1.1 equiv.) in acetic anhydride (11.7 mL, 123.4 mmol, 7.9 equiv.) was stirred at 160 ºC for 8 h. After completion of the reaction, cooled to room temperature, EtOAc (25 mL) and H_2O (15 mL) were added and the crude solution was concentrated to remove the excess acetic anhydride. EtOAc (80 mL) and 10% aqueous Na_2CO_3 (25 mL) were added to the crude and two layers separated. The aqueous layer was extracted with EtOAc (2 x 40 mL). The combined organic layer was washed with H_2O (2 x 35 mL) and brine (35 mL), dried over anhydrous sodium sulfate and concentrated *in vacuo*. A mixture of crude compound and K_2CO_3 (3.39 g, 24.528 mmol, 1.57 equiv.) in MeOH (20 mL) were refluxed for 1 h. After completion of the reaction, cool to room temperature and the solvent was removed under reduced pressure. The crude was neutralized with 3N HCl and extracted with EtOAc (2 x 75 mL). The combined organic layer was washed with H_2O (2 x 35 mL) and brine (35 mL), dried over anhydrous sodium sulfate and concentrated in vacuo. The crude was purified by column chromatography (EtOAc/hexane=1/5–1/4) to afford the desired product **1** (1.4 g, 43.6%, over 3 steps) as pale yellow color solid. R_f = 0.27 (EtOAc/hexane=1/4). ^{1}H NMR (400 MHz, $CDCl_3$) δ 7.81 (1H, t, *J* = 1.1 Hz), 7.67 (1H, d, *J* = 2.2 Hz), 7.59 (1H, d, *J* = 1.1 Hz), 7.11 (1H, br s), 6.94 (1H, dd, *J* = 2.2, 1.1 Hz), 4.42 (2H, q, *J* = 7.1 Hz), 1.41 (3H, t, *J* = 7.1 Hz); 13C NMR (100 MHz, CDCl3) δ 167.8, 156.1, 149.8, 146.5, 127.0, 121.5, 109.1, 106.2, 104.2, 61.7, 14.3; EI-MS *m/z* 206 ($[M]^+$), 191, 178, 161 (base).

Ethyl 4-hydroxybenzo[*b*]thiophene-6-carboxylate (2):

Following the method used for compound **1** preparation, the compound **2** was obtained from 2-thiophenecarboxaldehyde as off-white solid. Yield: 42.8%. R_f = 0.31 (ethyl acetate /hexane=1/4). ^{1}H NMR (400MHz, $CDCl_3$) δ 8.20 (1H, d, *J* = 1.2 Hz), 7.60 (1H, d, *J* = 1.2 Hz), 7.55 (2H, s), 6.55 (1H, s), 4.43 (2H, q, *J* = 7.2 Hz), 1.43 (3H, t, *J* = 7.2 Hz).

Ethyl 4-(ethoxymethoxy)benzofuran-6-carboxylate (3):

To a stirred solution of compound **1** (0.41 g, 1.969 mmol) in anhydrous acetone (8 mL) was added K_2CO_3 (0.41 g, 2.954 mmol) under nitrogen atmosphere, cooled to 0 ºC and stirred for 20 min. Chloromethyl ethyl ether (0.22 mL, 2.363 mmol) was added dropwise followed by tetrabutylammonium iodide (0.07 g, 0.197 mmol) in anhydrous acetone. The reaction mixture was warmed to room temperature and stirred for 15 h. After completion of the reaction, filtered through Celite® pad and washed with acetone (20 mL). The filtrate was concentrated *in vacuo*. The crude was purified by column chromatography (EtOAc/hexane=1/6) to afford the desired product **3** (0.45 g, 87.0%) as pale-brown solid. mp 58–60 ºC; R_f = 0.67 (EtOAc/hexane=1/4); ^{1}H NMR (400 MHz, $CDCl_3$): δ 7.91 (1H, t, *J* = 1.2 Hz), 7.67 (1H, d, *J* = 2.4 Hz), 7.60 (1H, d, *J* = 1.2 Hz), 6.92 (1H, dd, *J* = 2.4, 1.2 Hz), 5.40 (2H, s), 4.4 (2H, q, *J* = 7.2 Hz), 3.79 (2H, q, *J* = 7.2 Hz), 1.41 (3H, t, *J* = 7.2 Hz), 1.25 (3H, t, *J* = 7.2 Hz); ^{13}C NMR (100 MHz, $CDCl_3$): δ 166.8, 155.7, 150.6, 146.5, 127.7, 122.8, 108.1, 107.6, 104.4, 93.9, 64.8, 61.2, 15.2, 14.4; EI-MS *m/z* 264 ($[M]^+$), 219, 191, 84 (base).

Ethyl 4-(ethoxymethoxy)benzo[*b*]thiophene-6-carboxylate (4):

To a stirred suspension of NaH (60% in mineral oil; 0.04 g, 0.907 mmol, 1.3 equiv.) in anhydrous *N*,*N*-dimethylformamide (2 mL) was added compound **2** (0.16 g, 0.697 mmol, 1.0 equiv.) in anhydrous *N*,*N*-dimethylformamide (2 ml) slowly at 0 ºC under nitrogen atmosphere and stirred for 20 min. Chloromethyl ethyl ether (0.07 mL, 0.767 mmol, 1.1 equiv.) was slowly added to it. The resulting mixture was warmed to ambient temperature and stirred for 7 h. After completion of the reaction, quenched with H_2O (5 mL) and extracted with ethyl acetate (3 x 20 mL). The combined organic layer was washed with H_2O (3 x 15 mL) and brine (15 mL), dried over anhydrous sodium sulfate and concentrated *in vacuo*. The crude was purified by column chromatography (ethyl acetate/hexane=1/6) to afford the desired product **4** (0.17 g, 87.9%) as pale yellow color solid. $R_{f\,=}$ 0.70 (EtOAc/hexane=1/4). ^{1}H NMR (400MHz, $CDCl_3$) δ 8.27 (1H, d, *J* = 1.2 Hz), 7.65 (1H, d, *J* = 1.2 Hz), 7.54 (2H, s), 5.44 (2H, s), 4.41 (2H, q, *J* = 7.2 Hz), 3.80 (2H, q, *J* = 7.2 Hz), 1.42 (3H, t, *J* = 7.2 Hz), 1.25 (3H, t, *J* = 7.2 Hz).

Ethyl 4-methoxybenzofuran-6-carboxylate (5):

To a stirred solution of compound **1** (0.8 g, 3.88 mmol, 1.0 equiv.) in anhyd. DMF (10 mL) was added K_2CO_3 (0.7 g, 5.044 mmol, 1.3 equiv.) under argon atmosphere at ambient

temperature and the mixture was stirred for 30 min. Iodomethane (0.27 mL, 4.268 mmol, 1.1 equiv.) was added slowly to the above mixture and stirred for 4 h. After completion of the reaction, the reaction mixture was neutralized with 1N HCl and extracted with EtOAc (2 x 75 mL). The combined organic layer was washed with H_2O (3 x 35 mL) and brine (30 mL), dried over anhydrous Na_2SO_4 and concentrated *in vacuo*. The crude was purified by column chromatography (EtOAc/hexane=1/10) to afford the desired product **5** (0.81 g, 95.0%) as pale yellow solid. R_f = 0.66 (EtOAc/hexane=1/4); ^{1}H NMR (400 MHz, $CDCl_3$): δ 7.88 (1H, t, *J* = 0.8 Hz), 7.67 (1H, d, *J* = 2.0 Hz), 7.37 (1H, d, *J* = 1.2 Hz), 6.90 (1H, dd, *J* = 2.0, 1.2 Hz), 4.41 (2H, q, *J* = 7.2 Hz), 4.0 (3H, s), 1.42 (3H, t, *J* = 7.2 Hz); ^{13}C NMR (100 MHz, $CDCl_3$): δ 166.9, 155.5, 153.1, 146.3, 127.5, 122.0, 106.8, 104.3, 104.0, 61.1, 55.8, 14.4, ; EI-MS *m/z* 220 ($[M]^+$), 205, 192, 177 (base).

(4-(Ethoxymethoxy)benzofuran-6-yl)methanol (6):

To a stirred solution of compound **3** (0.51 g, 1.93 mmol) in anhyd. THF (9 mL) was added $LiAlH_4$ (1.0 M in THF, 2.9 mL) dropwise under nitrogen atmosphere at 0 ºC. The reaction mixture was warmed to room temperature and stirred for 2 h. After completion of the reaction, cool to 0 ºC, saturated aqueous Na_2SO_4 solution (4 mL) was added dropwise and stirred for 20 min at room temperature. The reaction mixture was filtered through Celite® pad and washed with EtOAc (30 mL). The filtrate was washed with H_2O (2 x 10 mL) and brine (10 mL), dried over anhydrous Na_2SO_4 and concentrated *in vacuo*. The crude was purified by column chromatography (EtOAc/hexane=1/3) to afford the desired product **6** (0.43 g, 98.0%) as colorless liquid. R_f = 0.21 (EtOAc/hexane=1/4); ^{1}H NMR (400 MHz, $CDCl_3$): δ 7.53 (1H, d, *J* = 2.4 Hz), 7.20 (1H, t, *J* = 0.8 Hz), 6.92 (1H, d, *J* = 0.8 Hz), 6.84 (1H, dd, *J* = 2.4, 0.8 Hz), 5.35 (2H, s), 4.75 (2H, s), 3.78 (2H, q, *J* = 7.2 Hz), 1.95 (1H, br s), 1.24 (3H, t, *J* = 7.2 Hz); ^{13}C NMR (100 MHz, $CDCl_3$): δ 156.4, 151.0, 144.1, 138.7, 117.9, 106.3, 104.0, 103.9, 93.5, 65.6, 64.6, 15.1; EI-MS *m/z* 222 ($[M]^+$, base), 192, 163, 147.

(4-(Ethoxymethoxy)benzo[*b*]thiophen-6-yl)methanol (7):

Following the method used for compound **6** preparation, reduction of compound **4** (1.3 g, 4.64 mmol, 1.0 equiv.) in anhyd. THF (18 mL) using $LiAlH_4$ (1.0 M in THF, 5.57 mL, 5.569 mmol, 1.2 equiv.) in 3 h afforded the compound **7** (1.07 g, 96.7%) as colorless liquid. R_f = 0.21 (EtOAc/hexane=1/4); ^{1}H NMR (400 MHz, $CDCl_3$): δ 7.52 (1H, t, *J* = 0.8 Hz), 7.47 (1H,

dd, *J* = 5.6, 0.8 Hz), 7.34 (1H, d, *J* = 5.6 Hz), 7.02 (1H, d, *J* = 0.8 Hz), 5.39 (2H, s), 4.78 (2H, d, *J* = 5.2 Hz), 3.79 (2H, q, *J* = 7.2 Hz), 1.79 (1H, br t, *J* = 5.6 Hz), 1.24 (3H, t, *J* = 7.2 Hz); ^{13}C NMR (100 MHz, $CDCl_3$): δ 152.6, 141.5, 138.7, 130.6, 125.1, 120.4, 114.0, 106.9, 93.3, 65.6, 64.6, 15.1; EI-MS *m/z* 238 ($[M]^+$, base), 208, 180, 163.

(4-Methoxybenzofuran-6-yl)methanol (8):

Following the method used for compound **6** preparation, reduction of compound **5** (0.16 g, 0.704 mmol, 1.0 equiv.) in anhyd. THF (4 mL) using $LiAlH_4$ $LiAlH_4$ (1.0 M in THF, 0.84 mL, 1.2 equiv.) in 3 h afforded the compound **8** (0.13 g, 97.0%) as colorless liquid. R_f = 0.18 (EtOAc/hexane=1/3); ^{1}H NMR (400 MHz, $CDCl_3$): δ 7.52 (1H, d, *J* = 2.0 Hz), 7.13 (1H, t, *J* = 0.8 Hz), 6.84 (1H, dd, *J* = 2.4, 0.8 Hz), 6.69 (1H, s), 4.76 (2H, s), 3.94 (3H, s), 1.87 (1H, br s); ^{13}C NMR (100 MHz, $CDCl_3$): δ 156.2, 153.6, 143.9, 138.6, 117.1, 103.9, 103.2, 102.4, 65.8, 55.6; EI-MS *m/z* 178 ($[M]^+$, base), 161, 149, 84.

4-(Ethoxymethoxy)benzofuran-6-carbaldehyde (9):

IBX (0.59 g, 2.092 mmol) was suspended in anhydrous DMSO (2 mL) and stirred for 5 min at room temperature under nitrogen atmosphere (clear solution formed). Compound **6** (0.31 g, 1.395 mmol) in anhydrous THF (2 mL) was added to above mixture. Compound **6** containing round bottom flask was rinsed with anhydrous DMSO (1 mL) and added to the reaction mixture. The reaction mixture was stirred for 2 h. After completion of the reaction, ice-cold H_2O (8 mL) and ether (15 mL) were added to the reaction mixture and stirred for 5 min. The reaction mixture was extracted with ether (3 x 25 mL). The combined organic layer was washed with H_2O (3 x 15 mL) and brine (15 mL), dried over anhydrous Na_2SO_4 and concentrated *in vacuo*. The crude was purified by column chromatography (EtOAc/hexane=1/5) to afford the desired product **9** (0.29 g, 95.4%) as white solid. mp 71–73 ºC; R_f = 0.61 (EtOAc/hexane=1/4); ^{1}H NMR (400 MHz, $CDCl_3$): δ 10.01 (1H, s), 7.74 (1H, d, *J* = 2.0 Hz), 7.70 (1H, t, *J* = 1.2 Hz), 7.46 (1H, d, *J* = 1.2 Hz), 6.95 (1H, dd, *J* = 2.0, 1.2 Hz), 5.42 (2H, s), 3.79 (2H, q, *J* = 7.2 Hz), 1.25 (3H, t, *J* = 7.2 Hz); ^{13}C NMR (100 MHz, $CDCl_3$): δ 191.7, 155.8, 151.5, 147.5, 134.4, 124.4, 108.8, 106.6, 104.8, 93.6, 65.0, 15.2; EI-MS *m/z* 220 ($[M]^+$, base), 190, 175, 161.

4-(Ethoxymethoxy)benzo[*b*]thiophene-6-carbaldehyde (10):

IBX (1.73 g, 6.169 mmol, 1.4 equiv.) was suspended in anhydrous DMSO (8 mL) and stirred for 5–10 min at room temperature under nitrogen atmosphere until clear solution was formed. Compound **7** (1.05 g, 4.406 mmol, 1.0 equiv.) in anhydrous DMSO (1.0 mL)/THF (3 mL) was added to above solution. Compound **7** containing round bottom flask was rinsed with anhydrous THF (1.5 mL) and added to the reaction mixture. The reaction mixture was stirred for 2.5 h. After completion of the reaction, ice-cold H_2O (15 mL) and ether (25 mL) were added to the reaction mixture and stirred for 10 min. The reaction mixture was filtered through Celite® pad and washed with EtOAc (50 mL). The filtrate was placed in 250 mL separating funnel and two layers separated. Aqueous layer was extracted with EtOAc (2 x 50 mL). The combined organic layer was washed with H_2O (3 x 35 mL) and brine (35 mL), dried over anhydrous Na_2SO_4 and concentrated *in vacuo*. The crude was purified by column chromatography (EtOAc/hexane=1/6) to afford the desired product **10** (0.99 g, 95.0%) as pale yellow solid. R_f = 0.49 (EtOAc/hexane=1/4); ^{1}H NMR (400 MHz, $CDCl_3$): δ 10.04 (1H, s), 8.03 (1H, s), 7.64 (1H, d, *J* = 5.2 Hz), 7.58 (1H, dd, *J* = 5.2, 1.2 Hz), 7.51 (1H, d, *J* = 1.2 Hz), 5.45 (2H, s), 3.80 (2H, q, *J* = 7.2 Hz), 1.25 (3H, t, *J* = 7.2 Hz); ^{13}C NMR (100 MHz, $CDCl_3$): δ 191.7, 153.0, 141.1, 135.8, 134.4, 130.1, 121.0, 120.8, 105.6, 93.4, 64.9, 15.1; EI-MS *m/z* 236 ($[M]^+$, base), 177, 149.

4-Methoxybenzofuran-6-carbaldehyde (11):

IBX (1.12 g, 3.952 mmol, 1.4 equiv.) was suspended in anhydrous DMSO (5 mL) and stirred for 5–10 min at room temperature under nitrogen atmosphere until clear solution was formed. Compound **8** (0.5 g, 2.823 mmol, 1.0 equiv.) in anhydrous DMSO (2.5 mL) was added to above solution. The reaction mixture was stirred for 3 h. After completion of the reaction, ice-cold H_2O (10 mL) and ether (15 mL) were added to the reaction mixture and stirred for 10 min. The reaction mixture was filtered through Celite® pad and washed with ether (25 mL). The filtrate was placed in 250 mL separating funnel and two layers separated. Aqueous layer was extracted with ether (2 x 40 mL). The combined organic layer was washed with H_2O (3 x 30 mL) and brine (30 mL), dried over anhydrous Na_2SO_4 and concentrated *in vacuo*. The crude was purified by column chromatography (EtOAc/hexane=1/8) to afford the desired product **11** (0.47 g, 94.0%) as white solid. R_f = 0.53 (EtOAc/hexane = 1/4); ^{1}H NMR (400 MHz, $CDCl_3$): δ 10.00 (s, 1H), 7.74 (d, *J* = 2.2 Hz, 1H), 7.64 (s, 1H), 7.23 (s, 1H), 6.94 (dd, *J* = 2.0, 0.8 Hz, 1H), 4.01 (s, 3H); ^{13}C NMR (100 MHz, $CDCl_3$): δ 191.7, 155.5, 154.0, 147.3,

134.4, 123.8, 109.6, 104.8, 101.4, 55.8; EI-MS *m/z* 176 ($[M]^+$, base), 175, 161, 147.

General procedure for Wittig-Horner reaction: To a stirred suspension of ^{t}BuOK (2.40 mmol, 1.20 equiv.) in anhyd. DMF (3.5 mL) was added triethyl phosphonoacetate (2.30 mmol, 1.15 equiv.) dropwise under nitrogen atmosphere at 0 ºC and the mixture was stirred for 30 min. Aldehyde (1.0 mmol) in anhydrous DMF (3.5 mL) was added slowly to the above mixture. The reaction mixture was warmed to room temperature and stirred for 4–5 h. After completion of the reaction, H_2O (5 mL) was added dropwise and stirred for 5 min and extracted with EtOAc (3 x 30 mL). The combined organic layer was washed with H_2O (3 x 20 mL) and brine (20 mL), dried over anhydrous Na_2SO_4 and concentrated *in vacuo*. The crude was purified by column chromatography (EtOAc/hexane=1/10–1/5) to afford the desired α,β-unsaturated ester.

Ethyl (*E*)-3-(4-(ethoxymethoxy)benzofuran-6-yl)acrylate (12): Yield: 90%; white solid. mp 46–48 ºC; R_f = 0.47 (EtOAc/hexane=1/5); ^{1}H NMR (400 MHz, $CDCl_3$): δ 7.75 (1H, d, *J* = 16.0 Hz), 7.60 (1H, d, *J* = 2.0 Hz), 7.35 (1H, t, *J* = 1.2 Hz), 7.13 (1H, d, *J* = 1.2 Hz), 6.87 (1H, dd, *J* = 2.0, 1.2 Hz), 6.45 (1H, d, *J* = 16.0 Hz), 5.37 (2H, s), 4.28 (2H, q, *J* = 7.2 Hz), 3.79 (2H, q, *J* = 7.2 Hz), 1.35 (3H, t, *J* = 7.2 Hz), 1.25 (3H, t, *J* = 7.2 Hz); ^{13}C NMR (100 MHz, $CDCl_3$): δ 167.2, 156.4, 151.3, 145.5, 145.2, 132.1, 120.7, 117.8, 106.6, 106.1, 104.5, 93.6, 64.7, 60.6, 15.2, 14.4; EI-MS *m/z* 290 ($[M]^+$, base), 260, 232, 215.

Ethyl (*E*)-3-(4-(ethoxymethoxy)benzo[*b*]thiophen-6-yl)acrylate (13): Yield: 94.5%; white solid. R_f = 0.55 (EtOAc/hexane=1/5); ^{1}H NMR (400 MHz, $CDCl_3$): δ 7.76 (1H, d, *J* = 16.0 Hz), 7.65 (1H, s), 7.48 (1H, dd, *J* = 5.6, 0.8 Hz), 7.43 (1H, dd, *J* = 5.6, 0.8 Hz), 7.23 (1H, s), 6.48 (1H, d, *J* = 16.0 Hz), 5.41 (2H, s), 4.28 (2H, q, *J* = 7.2 Hz), 3.79 (2H, q, *J* = 7.2 Hz), 1.35 (3H, t, *J* = 7.2 Hz), 1.25 (3H, t, *J* = 7.2 Hz); EI-MS *m/z* 306 ($[M]^+$, base), 276, 248, 236.

Ethyl (*E*)-3-(4-methoxybenzofuran-6-yl)acrylate (14): Yield: 86.2%; white solid. R_f = 0.54 (EtOAc/hexane=1/4); ^{1}H NMR (400 MHz, $CDCl_3$): δ 7.75 (1H, d, *J* = 16.0 Hz), 7.59 (1H, d, *J* = 2.4 Hz), 7.31 (1H, s), 6.86 (1H, dd, *J* = 2.4, 1.2 Hz), 6.83 (1H, s), 6.46 (1H, d, *J* = 16.0 Hz), 4.28 (2H, q, *J* = 7.2 Hz), 3.97 (3H, s), 1.35 (3H, t, *J* = 7.2 Hz); ^{13}C NMR (100 MHz, $CDCl_3$): δ 167.1, 156.2, 153.6, 145.3, 145.2, 131.9, 120.0, 117.6, 105.6, 104.4, 102.2, 60.5, 55.6, 14.4; EI-MS *m/z* 246 ($[M]^+$), 218, 201 (base), 174.

General procedure for α,β-unsaturated ester hydrolysis: To a stirred solution of α,β-unsaturated ester (1.0 mmol) in EtOH (5.0 mL) was added KOH (3–4 mmol) at room temperature. The reaction mixture was refluxed for 3–4 h. After completion of the reaction, cooled to room temperature and the solvent was removed under reduced pressure. The crude residue pH was adjusted to 6 with 1N HCl and extracted with CH_2Cl_2 (3 x 30 mL). The combined organic layer was washed with H_2O (3 x 20 mL) and brine (20 mL), dried over anhydrous Na_2SO_4 and concentrated *in vacuo*. The crude was purified by column chromatography ($MeOH/CH_2Cl_2$=1/50–1/20) to afford the desired α,β-unsaturated acid product.

(*E*)-3-(4-(Ethoxymethoxy)benzofuran-6-yl)acrylic acid (15): Yield: 92.0%; white solid. R_f = 0.30 (methanol/dichloromethane=1/20); ^{1}H NMR (400 MHz, $CDCl_3$): δ 7.86 (1H, d, *J* = 15.9 Hz), 7.62 (1H, d, *J* = 2.7 Hz), 7.38 (1H, s), 7.17 (1H, s), 6.89 (1H, dd, *J* = 2.0, 0.8 Hz), 6.48 (1H, d, *J* = 15.9 Hz), 5.39 (2H, s), 3.80 (2H, q, *J* = 7.1 Hz), 1.26 (3H, t, *J* = 7.3 Hz); ^{13}C NMR (100 MHz, $CDCl_3$): δ 172.4, 156.2, 151.3, 147.6, 145.7, 131.5, 121.1, 116.7, 106.7, 106.4, 104.5, 93.6, 64.7, 15.2; EI-MS *m/z* 262 ($[M]^+$, base), 232, 204, 187.

(*E*)-3-(4-(Ethoxymethoxy)benzo[*b*]thiophen-6-yl)acrylic acid (16): Yield: 70.5%; pale yellow solid. R_f = 0.24 (methanol/dichloromethane=1/50); ^{1}H NMR (400 MHz, $CDCl_3$): δ 7.87 (1H, d, *J* = 16.0 Hz), 7.68 (1H, s), 7.50 (1H, d, *J* = 5.6 Hz), 7.45 (1H, d, *J* = 5.6 Hz), 7.26 (1H, s), 6.51 (1H, *J* = 16.0 Hz), 5.42 (2H, s), 3.81 (2H, q, *J* = 7.2 Hz), 1.26 (3H, t, *J* = 7.2 Hz); ^{13}C NMR (100 MHz, $CDCl_3$): δ 172.3, 152.9, 147.4, 141.6, 133.2, 131.7, 127.6, 120.8, 117.8, 116.9, 106.2, 93.5, 64.7, 15.2; EI-MS *m/z* 278 ($[M]^+$, base), 248, 220, 203.

(*E*)-3-(4-Methoxybenzofuran-6-yl)acrylic acid (17): Yield: 50.8%; brown color solid. R_f = 0.54 (methanol/dichloromethane=1/20); ^{1}H NMR (400 MHz, DMSO-d_6): δ 12.38 (1H, br s), 8.00 (1H, d, *J* = 2.0 Hz), 7.69 (1H, d, *J* = 16.0 Hz), 7.59 (1H, s), 7.18 (1H, s), 6.97 (1H, dd, *J* = 2.0, 0.8 Hz), 6.66 (1H, *J* = 16.0 Hz), 3.96 (3H, s); ^{13}C NMR (100 MHz, DMSO-d_6): δ 167.8, 155.6, 153.2, 146.5, 144.6, 132.1, 119.2, 118.8, 105.6, 104.3, 103.1, 55.8; EI-MS *m/z* 218 ($[M]^+$, base), 201, 147.

3-(4-(Ethoxymethoxy)benzofuran-6-yl)propanoic acid (18):

To a stirred solution of α,β-unsaturated carboxylic acid (0.26 g, 1.0 mmol) in EtOAc (6 mL)

was added 10% Pd/C (0.02 g) and the reaction mixture was stirred under hydrogen atmosphere for 3 h. After completion of the reaction (checked by TLC), CH_2Cl_2 (5 mL) was added and stirred for 5 min. The reaction mixture was filtered through Celite® pad and washed with CH_2Cl_2 (10 mL). The filtrate was concentrated under reduced pressure and the resulted crude was purified by column chromatography (MeOH/CH_2Cl_2=1/50–1/20) to afford the desired acid product **18** (0.24 g, 92.0%) as white solid; mp 78–80 ºC; R_f = 0.34 (MeOH/CH_2Cl_2=1/20); ^{1}H NMR (400 MHz, $CDCl_3$): δ 7.50 (1H, d, *J* = 2.4 Hz), 7.04 (1H, s), 6.82 (1H, dd, *J* = 2.4, 0.8 Hz), 6.80 (1H, d, *J* = 0.8 Hz), 5.34 (2H, s), 3.78 (2H, q, *J* = 7.2 Hz), 3.04 (2H, t, *J* = 7.6 Hz), 2.72 (2H, t, *J* = 7.6 Hz), 1.24 (3H, t, *J* = 7.2 Hz); ^{13}C NMR (100 MHz, $CDCl_3$): δ 179.0, 156.4, 150.9, 143.6, 137.8, 116.7, 107.8, 105.1, 103.9, 93.4, 64.5, 36.0, 31.0, 15.1; EI-MS *m/z* 264 ($[M]^+$), 234, 206, 147 (base).

2-Bromo-1-(4-methoxyphenyl)ethanone (19):

To a stirred solution of 4-methoxyacetophenone (1.5 g, 10.0 mmol) in anhydrous $CHCl_3$ (50 mL) was added *p*-toluenesulfonic acid monohydrate (0.38 g, 2.0 mmol) followed by *N*-bromosuccinimide (2.14 g, 12.0 mmol) at room temperature. The reaction mixture was stirred for 27 h. After completion of the reaction, H_2O (20 mL) and extracted with CH_2Cl_2 (2 x 50 mL). The combined organic layer was washed with H_2O (2 x 20 mL) and brine (20 mL), dried over anhydrous Na_2SO_4 and concentrated *in vacuo*. The crude was purified by column chromatography (EtOAc/hexane=1/7–1/5) and then re-columned (hexane/CH_2Cl_2=2/1) to afford the pure product **19** (1.8 g, 79.0%) as white solid.

2-Bromo-1-(4-hydroxyphenyl)ethanone (20):

To a stirred solution of 4-hydroxyacetophenone (1.36 g, 10.0 mmol) in $CHCl_3$ (11 mL) was added $CuBr_2$ (4.47 g, 20.0 mmol) in EtOH (9 mL) at room temperature. The reaction mixture was vigorously refluxed for 3.5 h. After completion of the reaction, cooled to room temperature, filtered through Celite pad and washed with CH_2Cl_2 (20 mL). The filtrate was concentrated in vacuo. The crude was dissolved in EtOAc (100 mL) and washed with H_2O (3 x 20 mL) and brine (20 mL), dried over anhydrous Na_2SO_4 and concentrated in vacuo. The crude was purified by column chromatography (100% CH_2Cl_2) to afford the pure product **20** (1.5 g, 70%) as white solid. ^{1}H NMR (400 MHz, DMSO-d_6): δ 10.51 (1H, br s), 7.84 (2H, d, *J* = 8.8 Hz), 6.83 (2H, d, *J* = 8.8 Hz), 4.73 (2H, s).

General procedure for α-azidoacetophenone synthesis: To a stirred solution of NaN_3 (0.29 g, 4.4 mmol) in H_2O (4 mL) was added α-bromoacetophenone (4.0 mmol) in MeOH (8 mL) dropwise at 0 ºC and stirred for 2 h. The reaction mixture was warmed to room temperature and stirred for 2.5–3.5 h. After completion of the reaction, MeOH was removed under reduced pressure. The crude residue diluted with H_2O (10 mL) and extracted with EtOAc (2 x 40 mL). The combined organic layer was washed with H_2O (2 x 15 mL) and brine (15 mL), dried over anhydrous Na_2SO_4 and concentrated in vacuo. The crude was purified by column chromatography (EtOAc/hexane=1/5–1/3) to afford the pure α-azidoacetophenone as white solid.

2-Azido-1-(4-methoxyphenyl)ethanone (22): Yield: 94.0%; R_f = 0.42 (EtOAc/hexane=1/5); ^{1}H NMR (400 MHz, $CDCl_3$): δ 7.87 (d, *J* = 8.8 Hz, 2H), 6.94 (d, *J* = 8.8 Hz, 2H), 4.51 (s, 2H), 3.87 (s, 3H).

2-Azido-1-(4-hydroxyphenyl)ethanone (23): Yield: 98%; R_f = 0.20 (EtOAc/hexane=1/3); ^{1}H NMR (400 MHz, DMSO-d_6): δ 10.50 (1H, s), 7.82 (2H, d, *J* = 8.8 Hz), 6.87 (2H, d, *J* = 8.8 Hz), 4.76 (2H, s).

General procedure for α-aminocetophenone hydrotosylate salts (α-ketoammonium tosylate) synthesis: To a stirred solution of α-azidoacetophenone (4 mmol) in THF (20 mL) were added triphenylphosphine (1.5–2 equiv.) and *p*-TsOH (4 equiv.) at room temperature. The reaction mixture was stirred at 25–30 ºC for 20–24 h. After completion of the reaction, filtered and washed with cold THF to afford NMR-clean α-aminocetophenone hydrotosylate salts (α-ketoammonium tosylate), which were used in the next step without further purification.

2-(4-Methoxyphenyl)-2-oxoethan-1-aminium 4-methylbenzenesulfonate (25): Yield: 72.0%; ^{1}H NMR (400 MHz, DMSO-d_6): δ 8.16 (3H, br s), 8.00 (2H, d, *J* = 8.8 Hz), 7.47 (2H, d, *J* = 8.0 Hz), 7.11 (4H, d, *J* = 8.8 Hz), 4.55 (2H, s), 3.87 (3H, s), 2.29 (3H, s); ^{13}C NMR (100 MHz, DMSO-d_6): δ 191.1, 164.0, 145.4, 137.6, 130.5, 128.0, 126.4, 125.4, 114.1, 55.6, 44.4, 20.7.

2-(4-Hydroxyphenyl)-2-oxoethanaminium 4-methylbenzenesulfonate (26): Yield: 36.0%; ^{1}H NMR (400 MHz, DMSO-d_6): δ 10.58 (1H, br s), 8.12 (3H, br s), 7.89 (2H, d, *J* = 8.8 Hz),

7.48 (2H, d, *J* = 8.0 Hz), 7.11 (2H, d, *J* = 8.0 Hz), 6.91 (2H, d, *J* = 8.8 Hz), 4.48 (2H, s), 2.29 (3H, s); ^{13}C NMR (100 MHz, DMSO-d_6): δ 190.8, 163.2, 145.4, 137.8, 130.9, 128.1, 125.5, 125.2, 115.6, 44.3, 20.8.

2-Oxo-2-phenylethanaminium 4-methylbenzenesulfonate (27):

Yield: 54.0%; ^{1}H NMR (400 MHz, DMSO-d_6): δ 8.22 (3H, br s), 8.02 (2H, d, *J* = 8.0 Hz), 7.75 (1H, tt, *J* = 7.6, 1.2 Hz), 7.60 (2H, td, *J* = 7.6, 1.2 Hz), 7.60 (2H, m), 7.11 (2H, d, *J* = 8.0 Hz), 4.62 (2H, s), 2.28 (3H, s).

2-Bromo-1-(4-(ethoxymethoxy)phenyl)ethanone (28):

To a stirred solution of compound **20** (0.78 g, 3.63 mmol) in anhydrous CH_2Cl_2 (6 mL) was added *N*,*N*-diisopropylethylamine (0.82 mL, 4.72 mmol) under argon atmosphere at 0 ºC. After stirring for 15 min, chloromethyl ethyl ether (0.37 mL, 3.99 mmol) was added slowly. The reaction mixture was warmed to room temperature and stirred for 2 h. After completion of the reaction, H_2O (20 mL) was added and extracted with CH_2Cl_2 (2 x 40 mL). The combined organic layer was washed with H_2O (3 x 25 mL) and brine (25 mL), dried over anhydrous Na_2SO_4 and concentrated *in vacuo*. The crude was purified by column chromatography (EtOAc/hexane=1/8) to afford the pure product **28** (0.76 g, 77.2%) as colorless liquid. $R_{f\,=}$ 0.45 (EtOAc/hexane=1/4); ^{1}H NMR (400MHz, $CDCl_3$) δ 7.95 (2H, d, *J* = 8.4 Hz), 7.12 (2H, d, *J* = 8.4 Hz), 5.31 (2H, s), 4.68 (2H, s), 3.75 (2H, q, *J* = 7.2 Hz), 1.23 (3H, t, *J* = 7.2 Hz).

***S*-(2-(4-(Ethoxymethoxy)phenyl)-2-oxoethyl) ethanethioate (29):**

To a stirred solution of compound **28** (0.30 g, 1.09 mmol) in anhydrous tetrahydrofuran (8 mL) was added potassium thioacetate (0.16 g, 1.42 mmol) under argon atmosphere at room temperature and the resulting mixture was stirred for 4 h. After completion of the reaction, solvent was removed under reduced pressure. H_2O (20 mL) was added to the crude and extracted with EtOAc (2 x 40 mL). The combined organic layer was washed with H_2O (3 x 25 mL) and brine (25 mL), dried over anhydrous Na_2SO_4 and concentrated *in vacuo*. The crude was purified by column chromatography (EtOAc/hexane=1/8) to afford the pure product **29** (0.28 g, 96.5%) as white solid. $R_{f\,=}$ 0.52 (EtOAc/hexane=1/4); ^{1}H NMR (400MHz, $CDCl_3$) δ 7.97 (2H, d, *J* = 9.0 Hz), 7.09 1(2H, d, *J* = 9.0 Hz), 5.29 (2H, s), 4.37 (2H, s), 3.73

(2H, q, J = 7.0 Hz), 2.41 (3H, s), 1.22 (3H, t, J = 7.1 Hz)); ^{13}C NMR (100 MHz, $CDCl_3$) δ: 194.5, 192.0, 161.9, 130.9, 129.3, 116.0, 92.9, 64.8, 36.4, 30.4, 15.2; EI-MS *m/z* 268 ($[M]^+$), 179 (base), 121.

1-(4-(Ethoxymethoxy)phenyl)-2-mercaptoethan-1-one (30):

To a stirred solution of compound **29** (0.28 g, 1.04 mmol) in anhydrous MeOH (6 mL) was added NaOH (0.05 g, 1.35 mmol)) under argon atmosphere at room temperature and stirred for 1 h. After completion of the reaction, solvent was removed under reduced pressure. H_2O (15 mL) was added was added to the crude residue, PH was adjusted to 6–7 using 1N HCl and extracted with EtOAc (2 x 40 mL). The combined organic layer was washed with H_2O (3 x 30 mL) and brine (25 mL), dried over anhydrous Na_2SO_4 and concentrated *in vacuo*. The crude was purified by column chromatography (EtOAc/hexane=1/6) to afford the pure product **30** (0.19 g, 82.6%) as colorless liquid. R_f = 0.46 (EtOAc/hexane=1/4); ^{1}H NMR (400MHz, $CDCl_3$) δ 7.94 (2H, d, J = 8.9 Hz), 7.10 (2H, d, J = 8.9 Hz), 5.29 (2H, s), 3.92 (2H, d, J = 7.2 Hz), 3.74 (2H, q, J = 7.0 Hz), 2.14 (1H, t, J = 7.3 Hz), 1.22 (3H, t, J = 7.0 Hz); ^{13}C NMR (100Hz, $CDCl_3$) δ: 193.6, 161.8, 130.9, 128.8, 116.1, 92.9, 64.8, 31.0, 15.2; EI-MS *m/z* 226 ($[M]^+$), 194, 179, 121 (base).

General procedure for amide coupling: To a stirred solution of acid (0.4 mmol) and HATU (0.48 mmol, 1.2 equiv.) in anhydrous DMF (4 mL) was added *N*-methylmorpholine (NMM) (1.0 mmol, 2.5 equiv.) at 15-20 ºC and stirred for 15 min. under nitrogen atmosphere. α-Aminocetophenone hydrotosylate salt (0.44 mmol, 1.1 equiv.)/4-hydroxyphenethylamine (0.06 g, 0.44 mmol, 1.1 equiv.) was added to it and the reaction mixture was stirred for 5–9 h. After completion of the reaction, H_2O (8 mL) was added to it and then extracted with EtOAc (3 x 30 mL). The combined organic layer was washed with H_2O (3 x 15 mL) and brine (15 mL), dried over anhydrous Na_2SO_4 and concentrated *in vacuo*. The crude was purified by column chromatography ($MeOH/CH_2Cl_2$=1/50 – 1/20) to afford the pure product.

3-(4-(Ethoxymethoxy)benzofuran-6-yl)-*N*-(2-(4-methoxyphenyl)-2-oxoethyl)propanamide (31): Yield: 96.0%; White solid; mp 137–139 ºC; R_f = 0.50 (methanol/dichloromethane=1/20); ^{1}H NMR (400 MHz, $CDCl_3$): δ 7.95 (2H, d, J = 8.8 Hz), 7.49 (1H, d, J = 2.4 Hz), 7.06 (1H, s), 6.96 (2H, d, J = 8.8 Hz), 6.82 (1H, dd, J = 2.4, 0.8 Hz), 6.81 (1H, d, J = 0.8 Hz), 6.63 (1H, br s), 5.34 (2H, s), 4.72 (2H, d, J = 4.0 Hz), 3.89 (3H, s),

3.78 (2H, q, *J* = 7.2 Hz), 3.10 (2H, t, *J* = 7.6 Hz), 2.66 (2H, t, *J* = 7.6 Hz), 1.24 (3H, t, *J* = 7.2 Hz); ^{13}C NMR (100 MHz, $CDCl_3$): δ 192.5, 172.1, 164.2, 156.5, 150.9, 143.5, 138.5, 130.2, 127.2, 116.6, 114.1, 107.8, 105.1, 103.9, 93.5, 64.5, 55.6, 46.0, 38.5, 32.0, 15.2; EI-MS *m/z* 411 (M^+), 321, 216, 135 (base).

3-(4-(Ethoxymethoxy)benzofuran-6-yl)-*N*-(2-(4-hydroxyphenyl)-2-oxoethyl)propanamide (32):

Yield: 80.0%; white solid; mp 145–147 ºC; R_f = 0.50 (EtOAc/hexane = 2/1); ^{1}H NMR (400 MHz, DMSO-d_6): δ 10.41 (1H, s), 8.18 (1H, t, *J* = 5.6 Hz), 7.87 (2H, d, *J* = 8.8 Hz), 7.84 (1H, d, *J* = 2.0 Hz), 7.13 (1H, s), 6.89 (1H, dd, *J* = 2.0, 0.8 Hz), 6.87 (2H, d, *J* = 8.8 Hz), 6.82 (1H, d, *J* = 0.8 Hz), 5.36 (2H, s), 4.53 (2H, d, *J* = 5.6 Hz), 3.71 (2H, q, *J* = 7.2 Hz), 2.93 (2H, t, *J* = 7.8 Hz), 2.55 (2H, t, *J* = 7.8 Hz), 1.15 (3H, t, *J* = 7.2 Hz); ^{13}C NMR (100 MHz, DMSO-d_6): δ 193.3, 171.7, 162.3, 155.9, 150.2, 144.4, 139.1, 130.3, 126.6, 116.0, 115.3, 108.2, 104.6, 103.8, 93.1, 63.9, 45.2, 31.5, 15.1; EI-MS *m/z* 397 (M^+), 339, 216, 147 (base).

(*E*)-3-(4-(Ethoxymethoxy)benzofuran-6-yl)-*N*-(2-(4-methoxyphenyl)-2-oxoethyl)acrylamide (33): Yield: 92.0%; white solid; mp 153–155 ºC; R_f = 0.58 (methanol/dichloromethane=1/20); ^{1}H NMR (400 MHz, $CDCl_3$): δ 8.01 (2H, d, *J* = 8.8 Hz), 7.74 (1H, d, *J* = 15.6 Hz), 7.60 (1H, d, *J* = 2.0 Hz), 7.36 (1H, s), 7.16 (1H, d, *J* = 1.2 Hz), 6.98 (2H, d, *J* = 8.8 Hz), 6.87 (1H, dd, *J* = 2.0, 0.8 Hz), 6.86 (1H, t, *J* = 4.4 Hz), 6.59 (1H, d, *J* = 15.6 Hz), 5.39 (2H, s), 4.87 (2H, d, *J* = 4.4 Hz), 3.90 (3H, s), 3.80 (2H, q, *J* = 7.2 Hz), 1.27 (3H, t, *J* = 7.2 Hz); ^{13}C NMR (100 MHz, $CDCl_3$): δ 192.6, 165.9, 164.3, 156.3, 151.2, 145.2, 141.9, 132.2, 130.3, 127.3, 120.2, 119.7, 114.1, 106.3, 105.9, 104.4, 93.6, 64.6, 55.6, 46.3, 15.2; EI-MS *m/z* 409 (M^+), 274, 186 (base), 135.

(*E*)-3-(4-(Ethoxymethoxy)benzofuran-6-yl)-*N*-(2-(4-hydroxyphenyl)-2-oxoethyl)acrylamide (34): Yield: 71.0%; white solid; mp 206–208 ºC; R_f = 0.30 (EtOAc/hexane = 2/1); ^{1}H NMR (400 MHz, DMSO-d_6): δ 10.44 (1H, s), 8.40 (1H, t, *J* = 5.6 Hz), 8.00 (1H, d, *J* = 2.0 Hz), 7.92 (2H, d, *J* = 8.8 Hz), 7.54 (1H, d, *J* = 16.0 Hz), 7.52 (1H, s), 7.22 (1H, d, *J* = 0.8 Hz), 6.99 (1H, dd, *J* = 2.0, 0.8 Hz), 6.90 (2H, d, *J* = 8.8 Hz), 6.88 (1H, d, *J* = 16.0 Hz), 5.45 (2H, s), 4.70 (2H, d, *J* = 5.6 Hz), 3.75 (2H, q, *J* = 7.2 Hz), 1.17 (3H, t, *J* = 7.2 Hz); ^{13}C NMR (100 MHz, DMSO-d_6): δ 193.1, 165.2, 162.3, 155.8, 150.5, 146.3, 139.4, 132.4, 130.3, 126.5, 121.6, 119.4, 115.3, 104.2, 93.0, 64.0, 45.5, 15.0; EI-MS *m/z* 395 (M^+),

215, 186 (base), 121.

(*E*)-3-(4-(Ethoxymethoxy)benzofuran-6-yl)-*N*-(2-oxo-2-phenylethyl)acrylamide (35):

Yield: 93.5%; pale yellow solid; R_f = 0.53 (methanol/dichloromethane=1/20). ^{1}H NMR (400MHz, DMSO-d_6) δ 8.50 (br t, *J* = 5.6 Hz), 8.04 (2H, dd, *J* = 8.4, 1.6 Hz), 8.0 (1H, d, *J* = 2.4 Hz), 7.69 (1H, tt, *J* = 7.2, 1.2 Hz), 7.59–7.53 (4H, m), 7.22 (1H, s), 7.0 (1H, dd, *J* = 2.0, 0.8 Hz), 6.88 (1H, d, *J* = 15.6 Hz), 5.45 (2H, s), 4.80 (2H, d, *J* = 5.6 Hz), 3.75 (2H, q, *J* = 7.2 Hz), 1.17 (3H, t, *J* = 7.2 Hz); ^{13}C NMR (100 MHz, DMSO-d_6): δ 195.3, 165.4, 155.8, 150.6, 146.4, 139.6, 135.0, 133.6, 132.5, 128.9, 127.9, 121.5, 119.5, 105.9, 105.6, 104.3, 93.1, 64.1, 46.2, 15.1; EI-MS *m/z* 379 ($[M]^+$), 215, 186 (base), 105.

(*E*)-3-(4-(Ethoxymethoxy)benzo[*b*]thiophen-6-yl)-*N*-(2-(4-hydroxyphenyl)-2-oxoethyl)acrylamide (36):

Yield: 32.0%; pale yellow solid; R_f = 0.45 (EtOAc/hexane=2/1); ^{1}H NMR (400MHz, DMSO-d_6) δ 10.45 (1H, br s), 8.43 (1H, br t, *J* = 5.6 Hz), 7.92 (2H, d, *J* = 8.8 Hz), 7.87 (1H, s), 7.77 (1H, d, *J* = 5.6 Hz), 7.54 (1H, d, *J* = 15.6 Hz), 7.48 (1H, d, *J* = 5.6 Hz), 7.31 (1H, s), 6.89 (1H, d, *J* = 15.6 Hz), 6.88 (2H, d, *J* = 8.8 Hz), 5.48 (2H, s), 4.69 (2H, d, *J* = 5.6 Hz), 3.76 (2H, q, *J* = 7.2 Hz), 1.17 (3H, t, *J* = 7.2 Hz); ^{13}C NMR (100 MHz, DMSO-d_6): δ 193.2, 165.3, 162.4, 152.1, 141.1, 139.3, 132.6, 131.8, 130.4, 128.1, 126.5, 121.8, 120.3, 117.0, 115.4, 105.7, 93.0, 64.2, 45.6, 15.1; EI-MS *m/z* 411 ($[M]^+$), 202 (base), 179, 121.

(*E*)-*N*-(2-(4-Hydroxyphenyl)-2-oxoethyl)-3-(4-methoxybenzofuran-6-yl)acrylamide (TA15):

Yield: 28.3%; brown color solid; R_f = 0.41 (methanol/dichloromethane = 1/20). ^{1}H NMR (400MHz, DMSO-d_6) δ 10.46 (1H, br s), 8.31 (1H, br t, *J* = 5.2 Hz), 7.98 (1H, d, *J* = 1.6 Hz), 7.91 (2H, d, *J* = 8.4 Hz), 7.55 (1H, d, *J* = 15.6 Hz), 7.48 (1H, s), 7.07 (1H, s), 6.97 (1H, s), 6.93 (1H, d, *J* = 15.6 Hz), 6.88 (2H, d, *J* = 8.4 Hz), 4.69 (2H, d, *J* = 5.2 Hz), 3.97 (3H, s); ^{13}C NMR (100 MHz, DMSO-d_6): δ 193.2, 165.4, 162.4, 155.7, 153.1, 146.1, 139.6, 132.7, 130.4, 126.5, 121.6, 118.6, 115.4, 104.7, 104.3, 102.6, 55.6, 45.6; EI-MS *m/z* 351 ($[M]^+$), 202, 201 (base), 121; HRMS (EI) *m/z* (M^+) calcd for $C_{20}H_{17}NO_5$: 351.1107, Found: 351.1105.

(*E*)-3-(4-(Ethoxymethoxy)benzofuran-6-yl)-*N*-(4-hydroxyphenethyl)acrylamide (37):

Yield: 91.3%; pale yellow solid; R_f = 0.39 (methanol/dichloromethane=1/20). ^{1}H NMR (400MHz, DMSO-*d*$_6$) δ 9.20 (1H, br s), 8.18 (br t, *J* = 5.6 Hz), 8.0 (1H, d, *J* = 2.0 Hz), 7.49 (1H, d, *J* = 15.6 Hz), 7.48 (1H, s), 7.16 (1H, s), 7.03 (2H, d, *J* = 8.4 Hz), 6.98 (1H, dd, *J* = 2.4, 0.8 Hz), 6.69 (2H, d, *J* = 8.4 Hz), 6.64 (1H, d, *J* = 15.6 Hz), 5.43 (2H, s), 3.73 (2H, q, *J* = 7.2 Hz), 3.36 (2H, q, *J* = 7.2 Hz), 2.67 (2H, t, *J* = 7.2 Hz), 1.16 (3H, t, *J* = 7.2 Hz); ^{13}C NMR (100 MHz, DMSO-*d*$_6$): δ 164.9, 155.8, 155.7, 150.6, 146.4, 138.9, 132.6, 129.5, 122.0, 119.3, 115.1, 105.8, 105.5, 105.3, 93.1, 64.1, 40.7, 34.4, 15.1; EI-MS *m/z* 381 ([M]$^+$), 261 (base), 187, 120.

(*E*)-3-(4-(Ethoxymethoxy)benzo[*b*]thiophen-6-yl)-*N*-(4-hydroxyphenethyl)acrylamide (38):

Yield: 94.0%; pale yellow solid; R_f = 0.41 (methanol/dichloromethane=1/20). ^{1}H NMR (400MHz, DMSO-*d*$_6$) δ 9.19 (1H, s), 8.19 (1H, br t, *J* = 5.6 Hz), 7.82 (1H, s), 7.76 (1H, d, *J* = 5.6 Hz), 7.49 (1H, d, *J* = 16.0 Hz), 7.46 (1H, d, *J* = 5.6 Hz), 7.24 (1H, s), 7.03 (2H, d, *J* = 8.4 Hz), 6.69 (2H, d, *J* = 8.4 Hz), 6.66 (1H, d, *J* = 15.6 Hz), 5.46 (2H, s), 3.74 (2H, q, *J* = 7.2 Hz), 3.36 (2H, q, *J* = 7.2 Hz), 2.67 (2H, t, *J* = 7.2 Hz), 1.16 (3H, t, *J* = 7.2 Hz); ^{13}C NMR (100 MHz, DMSO-*d*$_6$): δ 164.9, 155.7, 152.1, 141.1, 138.8, 132.6, 131.7, 129.5, 128.0, 122.1, 120.3, 116.8, 115.1, 105.5, 93.0, 64.1, 40.7, 34.4, 15.1; EI-MS *m/z* 397 ([M]$^+$), 277 (base), 202, 120.

(*E*)-2-(4-Hydroxyphenyl)-2-oxoethyl 3-(4-(ethoxymethoxy)benzofuran-6-yl)acrylate (39):

To a mixture of acid **15** (0.11 g, 0.419 mmol, 1.0 equiv.), 2-bromo-1-(4-hydroxyphenyl)ethanone (**20**) (0.09 g, 0.419 mmol, 1.0 equiv.) and K_2CO_3 (0.07 g, 0.503 mmol, 1.2 equiv.) was added anhydrous DMF (4 mL) under argon atmosphere at ambient temperature and stirred the reaction mixture for 3 h. After completion of the reaction, H_2O (20 mL) was added, neutralized with 1N HCl and extracted with EtOAc (2 x 50 mL). The combined organic layer was washed with H_2O (3 x 25 mL) and brine (25 mL), dried over anhydrous Na_2SO_4 and concentrated *in vacuo*. The crude was purified by column chromatography (EtOAc/hexane=1/3–1/2) to afford the pure ester **39** (0.12 g, 73.2%) as white solid. R_f = 0.34 (EtOAc/hexane=2/3); ^{1}H NMR (400MHz, DMSO-*d*$_6$) δ 10.52 (1H, br s), 8.04 (1H, d, *J* = 2.4 Hz), 7.89 (2H, d, *J* = 8.8 Hz), 7.80 (1H, d, *J* = 16.0 Hz), 7.74 (1H, s),

7.35 (1H, s), 7.01 (1H, dd, *J* = 2.0, 0.8 Hz), 6.90 (2H, d, *J* = 8.8 Hz), 6.85 (1H, d, *J* = 16.0 Hz), 5.53 (2H, s), 5.47 (2H, s), 3.74 (2H, q, *J* = 7.2 Hz), 1.16 (3H, t, *J* = 7.2 Hz); ^{13}C NMR (100 MHz, DMSO-d_6): δ 190.7, 165.8, 162.7, 155.8, 150.5, 147.0, 145.7, 131.5, 130.4, 125.5, 120.5, 117.2, 115.5, 106.9, 106.4, 104.4, 93.1, 66.2, 64.2, 15.1; EI-MS *m/z* 396 ([M]$^+$), 262, 187, 121 (base).

(*E*)-*S*-(2-(4-(Ethoxymethoxy)phenyl)-2-oxoethyl) 3-(4-(ethoxymethoxy)benzofuran-6-yl)prop-2-enethioate (40):

To a stirred solution of carboxylic acid **15** (0.08 g, 0.3 mmol, 1.0 equiv.) in anhydrous dichloromethane (4 mL) were added 1-ethyl-3-(3-dimethylaminopropyl)carbodiimide (EDCI) (0.06 mL, 0.36 mmol, 1.2 equiv.) and 4-dimethylaminopyridine (DMAP) (0.01g, 0.06 mmol, 0.2 equiv.) under argon atmosphere and stirred for 20 min at room temperature. Thiol compound **30** (0.08 g, 0.36 mmol, 1.2 equiv.) in anhydrous dichloromethane (3 mL) was added and stirred for 2 h. After completion of the reaction, H_2O (20 mL) was added and extracted with dichloromethane (3 x 30 mL). The combined organic layer was washed with H_2O (3 x 25 mL) brine (25 mL), dried over anhydrous Na_2SO_4 and concentrated *in vacuo*. The crude was purified by column chromatography (EtOAc/hexane=1/6–1/4) to obtain the pure product **40** (0.1 g, 71.4%) as pale yellow solid; $R_{f\,=}$ 0.64 (EtOAc/hexane=1/2); ^{1}H NMR (400 MHz, $CDCl_3$) 8.02 (2H, d, *J* = 9.0 Hz), 7.73 (1H, d, *J* = 15.7 Hz), 7.62 (1H, d, *J* = 2.1 Hz), 7.37 (1H ,s), 7.14 (1H, s), 7.11 (2H, d, *J* = 8.9 Hz), 6.88 (1H, dd, *J* = 2.2, 0.9 Hz), 6.78 (1H, d, *J* = 15.7 Hz), 5.38 (2H, s), 5.30 (2H, s), 4.50 (2H, s), 3.79 (2H, q, *J* = 7.3 Hz), 3.74 (2H, q, *J* = 7.3 Hz), 1.26 (3H, t, *J* = 7.1 Hz), 1.22 (3H, t, *J* = 7.1 Hz); ^{13}C NMR (100Hz, $CDCl_3$) δ: 192.3, 188.2, 161.9, 156.4, 151.4, 145.9, 142.4, 131.5, 131.0, 129.5, 123.8, 121.4, 116.1, 106.8, 106.8, 104.7, 93.7, 92.9, 64.8, 36.2, 15.3, 15.2; EI-MS *m/z* (%): 470 ([M]$^+$), 245, 187, 179 (base).

General procedure for EOM-deprotection of amides

To a stirred solution/suspension of EOM-protected amide (0.25 mmol) in MeOH (6–12 mL) was added 1N HCl (0.75 mL) at room temperature and the mixture was stirred at 55–60 ºC for 1h. After completion of the reaction, cooled to room temperature and the solvent was removed under reduced pressure. H_2O (6 mL) was added to the crude, neutralized with aqueous saturated $NaHCO_3$ solution and extracted with EtOAc (3 x 35 mL). The combined

organic layer was washed with H_2O (2 x 20 mL), brine (20 mL), dried over anhydrous Na_2SO_4 and concentrated *in vacuo*. The crude was purified by column chromatography (EtOAc/hexane=2/1 and/or MeOH/CH_2Cl_2=1/50–1/20) to obtain the pure phenolic amide.

(*E*)-3-(4-hydroxybenzofuran-6-yl)-*N*-(2-(4-hydroxyphenyl)-2-oxoethyl)acrylamide (TA0): Yield: 63.0%; white solid. mp 257–259 ºC; R_f = 0.18 (MeOH/CH_2Cl_2 = 1/20); ^1H NMR (400 MHz, DMSO-d_6): δ 10.44 (1H, br s), 10.18 (1H, br s), 8.41 (1H, t, *J* = 5.6 Hz), 7.92 (2H, d, *J* = 8.8 Hz), 7.91 (1H, d, *J* = 2.0 Hz), 7.47 (1H, d, *J* = 15.6 Hz), 7.31 (1H, s), 6.99 (1H, dd, *J* = 2.0, 0.8 Hz), 6.89 (2H, d, *J* = 8.8 Hz), 6.88 (1H, s), 6.78 (1H,d, *J* = 15.6 Hz), 4.69 (2H, d, *J* = 5.6 Hz); ^{13}C NMR (100 MHz, DMSO-d_6): δ 193.3, 165.4, 162.4, 156.2, 151.2, 145.4, 139.7, 132.4, 130.4, 126.6, 121.0, 118.1, 115.4, 106.2, 104.5, 103.1, 45.5; EI-MS *m/z* 337 (M^+), 187 (base), 159, 121; HRMS (EI) *m/z* (M^+) calcd for $C_{19}H_{15}NO_5$: 337.0950, Found: 337.0950.

(*E*)-3-(4-hydroxybenzofuran-6-yl)-*N*-(2-(4-methoxyphenyl)-2-oxoethyl)acrylamide (TA11): Yield: 68.8%; Off-white solid. mp 210–212 ºC; R_f = 0.32 (methanol/dichloromethane=1/20); ^1H NMR (400 MHz, DMSO-d_6): δ 10.18 (1H, s), 8.42 (1H, br t, *J* = 5.6 Hz), 8.0 (2H, d, *J* = 8.8 Hz), 7.89 (1H, d, *J* = 2.0 Hz), 7.475 (1H, d, *J* = 16.0 Hz), 7.29 (1H, s), 7.07 (2H, d, *J* = 8.8 Hz), 6.97 (1H, d, *J* = 1.2 Hz), 6.87 (1H, s), 6.75 (1H, d, *J* = 16.0 Hz), 4.71 (2H, d, *J* = 5.6 Hz), 3.85 (3H, s); ^{13}C NMR (100 MHz, DMSO-d_6): δ 193.6, 165.4, 163.4, 156.2, 151.2, 145.4, 139.8, 132.4, 130.2, 127.9, 121.0, 118.1, 114.0, 106.2, 104.5, 103.1, 55.6, 45.7; EI-MS *m/z* 351 (M^+), 202, 187, 135 (base); HRMS (EI) *m/z* (M^+) calcd for $C_{20}H_{17}NO_5$: 351.1107, Found: 351.1110.

3-(4-Hydroxybenzofuran-6-yl)-*N*-(2-(4-hydroxyphenyl)-2-oxoethyl)propanamide (TA12): Yield: 89.4%; white solid; mp 209–211 ºC; R_f = 0.28 (methanol/dichloromethane=1/20); ^1H NMR (400 MHz, DMSO-d_6): δ 10.40 (1H, br s), 9.82 (1H, br s), 8.16 (1H, t, *J* = 5.6 Hz), 7.87 (2H, d, *J* = 8.8 Hz), 7.74 (1H, d, *J* = 2.4 Hz), 6.90 (1H, s), 6.88 (1H, dd, *J* = 2.4, 0.8 Hz), 6.86 (2H, d, *J* = 8.8 Hz), 6.49 (1H, d, *J* = 0.8 Hz), 4.51 (2H, d, *J* = 5.6 Hz), 2.85 (2H, t, *J* = 7.8 Hz), 2.51 (2H, t, *J* = 7.8 Hz); ^{13}C NMR (100 MHz, DMSO-d_6): δ 193.3, 171.8, 162.3, 156.3, 150.7, 143.4, 139.1, 130.3, 126.6, 115.3, 114.5, 108.4, 104.1, 102.0, 45.3, 37.1, 31.4; EI-MS *m/z* 339 (M^+, base), 160, 147, 121; HRMS (EI) *m/z* (M^+) calcd for $C_{19}H_{17}NO_5$: 339.1107, Found: 339.1110.

3-(4-Hydroxybenzofuran-6-yl)-*N*-(2-(4-methoxyphenyl)-2-oxoethyl)propanamide (TA13): Yield: 90.3%; off-white solid; mp 185–187 ºC; R_f = 0.36 (methanol/dichloromethane=1/20); ^{1}H NMR (400 MHz, DMSO-d_6): δ 9.81 (1H, s), 8.19 (1H, t, *J* = 5.6 Hz), 7.96 (2H, d, *J* = 8.8 Hz), 7.73 (1H, d, *J* = 2.4 Hz), 7.04 (2H, d, *J* = 8.8 Hz), 6.89 (1H, s), 6.88 (1H, dd, *J* = 2.4, 1.2 Hz), 6.49 (1H, d, *J* = 1.2 Hz), 4.54 (2H, d, *J* = 5.6 Hz), 3.84 (3H, s), 2.84 (2H, t, *J* = 7.2 Hz), 2.49 (2H, t, *J* = 7.2 Hz); ^{13}C NMR (100 MHz, DMSO-d_6): δ 193.7, 171.8, 163.3, 156.3, 150.7, 143.4, 139.1, 130.1, 127.9, 114.5, 114.0, 108.4, 104.1, 102.0, 55.6, 45.4, 37.1, 31.4; EI-MS *m/z* 353 (M^+, base), 160, 147, 135; HRMS (EI) *m/z* (M^+) calcd for $C_{20}H_{19}NO_5$: 353.1263, Found: 353.1261.

(*E*)-3-(4-Hydroxybenzofuran-6-yl)-*N*-(2-oxo-2-phenylethyl)acrylamide (TA14):

Yield: 92.0%; pale yellow solid; R_f = 0.31 (methanol/dichloromethane=1/20). ^{1}H NMR (400MHz, DMSO-d_6) δ 10.21 (1H, s), 8.51 (1H, t, *J* = 5.6 Hz), 8.04 (2H, d, *J* = 7.6 Hz), 7.91 (1H, d, *J* = 2.4 Hz), 7.69 (1H, t, *J* = 7.6 Hz), 7.57 (2H, d, *J* = 7.6 Hz), 7.48 (1H, d, *J* = 15.6 Hz), 7.32 (1H, s), 6.99 (1H, dd, *J* = 2.4, 1.2 Hz), 6.89 (1H, s), 6.78 (1H, d, *J* = 15.6 Hz), 4.79 (2H, d, *J* = 5.6 Hz); ^{13}C NMR (100 MHz, DMSO-d_6): δ 195.4, 165.5, 156.2, 151.2, 145.4, 139.9, 135.0, 133.6, 132.4, 128.9, 127.9, 120.9, 118.2, 106.2, 104.6, 103.2, 46.2; EI-MS *m/z* 321 ([M]$^+$), 187, 185, 105 (base); HRMS (EI) *m/z* (M^+) calcd for $C_{19}H_{15}NO_4$: 321.1001, Found: 321.1003.

(*E*)-2-(4-Hydroxyphenyl)-2-oxoethyl 3-(4-hydroxybenzofuran-6-yl)acrylate (TA21):

Yield: 82.4%; pale yellow solid; R_f = 0.31 (EtOAc/hexane=1/1). ^{1}H NMR (400MHz, DMSO-d_6) δ 10.52 (1H, br s), 10.23 (1H, br s), 7.95 (1H, d, *J* = 2.0 Hz), 7.88 (2H, d, *J* = 8.8 Hz), 7.74 (1H, d, *J* = 16.0 Hz), 7.55 (1H, s), 7.02 (1H, dd, *J* = 2.0, 0.8 Hz), 6.96 (1H, d, *J* = 0.8 Hz), 6.90 (2H, d, *J* = 8.8 Hz), 6.68 (1H, d, *J* = 16.0 Hz), 5.52 (2H, s); ^{13}C NMR (100 MHz, DMSO-d_6): δ 190.8, 165.8, 162.7, 156.1, 151.2, 146.0, 131.4, 130.4, 125.5, 119.2, 116.5, 115.5, 107.5, 104.6, 103.6, 66.1; EI-MS *m/z* 338 ([M]$^+$), 204, 187, 121 (base); HRMS (EI) *m/z* (M^+) calcd for $C_{19}H_{14}O_6$: 338.0790, Found: 338.0789.

(*E*)-*S*-(2-(4-Hydroxyphenyl)-2-oxoethyl) 3-(4-hydroxybenzofuran-6-yl)prop-2-enethioate (TA22):

Yield: 67.2%; pale yellow solid; R_f = 0.35 (methanol/dichloromethane=1/20). ^{1}H NMR (400

MHz, Acetone-d_6) δ: 9.30 (2H ,s), 8.00 (2H, d, J = 8.7 Hz), 7.84 (1H, d, J = 2.1 Hz), 7.69 (1H, d, J = 15.7 Hz), 7.51 (1H, s), 7.05 (1H, d, J = 1.5 Hz), 7.00 (1H, d, J = 2.2 Hz), 6.97 (2H, d, J = 8.7 Hz), 6.93 (1H, d, J = 15.8 Hz), 4.55 (2H, s); ^{13}C NMR (100Hz, DMSO-d_6) δ: 191.3, 187.9, 162.5, 156.1, 151.2, 146.2, 141.8, 131.1, 131.1, 127.1, 123.6, 119.5, 115.4, 107.9, 104.7, 104.0, 36.0; EI-MS m/z (%): 354 ([M]$^+$), 187, 121 (base); HRMS m/z (M$^+$) calcd for $C_{19}H_{14}O_5S$: 354.0562, Found: 354.0563.

(*E*)-3-(4-Hydroxybenzofuran-6-yl)-*N*-(4-hydroxyphenethyl)acrylamide (TA23):

Yield: 92.3%; pale yellow solid; R_f = 0.26 (methanol/dichloromethane=1/20). ^{1}H NMR (400MHz, DMSO-d_6) δ 10.18 (1h, br s), 9.20 (1H, br s), 8.16 (1H, br t, J = 5.6 Hz), 7.90 (1H, d, J = 2.0 Hz), 7.42 (1H, d, J = 15.6 Hz), 7.26 (1H, s), 7.03 (2H, d, J = 8.4 Hz), 6.98 (1H, dd, J = 2.0, 1.0 Hz), 6.83 (1H, d, J = 1.2 Hz), 6.69 (2H, d, J = 8.4 Hz), 6.56 (1H, d, J = 15.6 Hz), 3.35 (2H, q, J = 7.2 Hz), 2.67 (2H, t, J = 7.2 Hz); ^{13}C NMR (100 MHz, DMSO-d_6): δ 165.0, 156.2, 155.7, 151.2, 145.4, 139.2, 132.5, 129.5, 121.4, 118.0, 115.2, 106.1, 104.5, 103.0, 40.8, 34.4; EI-MS m/z 323 ([M]$^+$), 202 (base), 187, 120; HRMS (EI) m/z (M$^+$) calcd for $C_{19}H_{17}NO_4$: 323.1158, Found: 323.1157.

(*E*)-3-(4-Hydroxybenzo[*b*]thiophen-6-yl)-*N*-(2-(4-hydroxyphenyl)-2-oxoethyl)acrylamide (TA24):

Yield: 71.2%; pale yellow solid; R_f = 0.21 (methanol/dichloromethane=1/20). ^{1}H NMR (400MHz, DMSO-d_6) δ 10.44 (1H, br s), 10.26 (1H, br s), 8.46 (1H, t, J = 5.6 Hz), 7.92 (2H, d, J = 8.8 Hz), 7.67 (1H, d, J = 5.6 Hz), 7.66 (1H, s), 7.49 (1H, dd, J = 5.6, 0.8 Hz), 7.48 (1H, d, J = 15.6 Hz), 6.99 (1H, d, J = 0.8 Hz), 6.89 (2H, d, J = 8.8 Hz), 6.78 (1H, d, J = 15.6 Hz), 4.69 (2H, d, J = 5.6 Hz); ^{13}C NMR (100 MHz, DMSO-d_6): δ 193.3, 165.4, 162.4, 152.7, 141.3, 139.6, 132.7, 130.8, 130.4, 126.8, 126.6, 121.2, 120.7, 115.4, 114.7, 106.1, 45.6; EI-MS m/z 353 ([M]$^+$), 203 (base), 175, 121; HRMS (EI) m/z (M$^+$) calcd for $C_{19}H_{15}NO_4S$: 353.0722, Found: 353.0720.

(*E*)-3-(4-Hydroxybenzo[*b*]thiophen-6-yl)-*N*-(4-hydroxyphenethyl)acrylamide (TA25):

Yield: 94.5%; pale yellow solid; R_f = 0.27 (EtOAc/hexane=2/1); ^{1}H NMR (400MHz, DMSO-d_6) δ 10.16 (1H, s), 9.15 (1H, s), 8.14 (1H, br t, J = 5.6 Hz), 7.61 (1H, d, J = 5.6 Hz), 7.58 (1H, s), 7.43 (1H, dd, J = 5.6, 0.8 Hz), 7.39 (1H, d, J = 15.6 Hz), 6.99 (2H, d, J = 8.4 Hz),

6.89 (1H, d, *J* = 0.8 Hz), 6.65 (2H, d, *J* = 8.4 Hz), 6.52 (1H, d, *J* = 15.6 Hz), 3.30 (2H, q, *J* = 7.2 Hz), 2.63 (2H, t, *J* = 7.2 Hz); ^{13}C NMR (100 MHz, DMSO-d_6): δ 164.9, 155.7, 152.7, 141.3, 139.0, 132.7, 130.7, 129.5, 126.6, 121.6, 120.7, 115.1, 114.5, 106.1, 40.8, 34.4; EI-MS *m/z* 339 ([M]$^+$), 219 (base), 203, 120; HRMS (EI) *m/z* (M$^+$) calcd for $C_{19}H_{17}NO_3S$: 339.0929, Found: 339.0929.

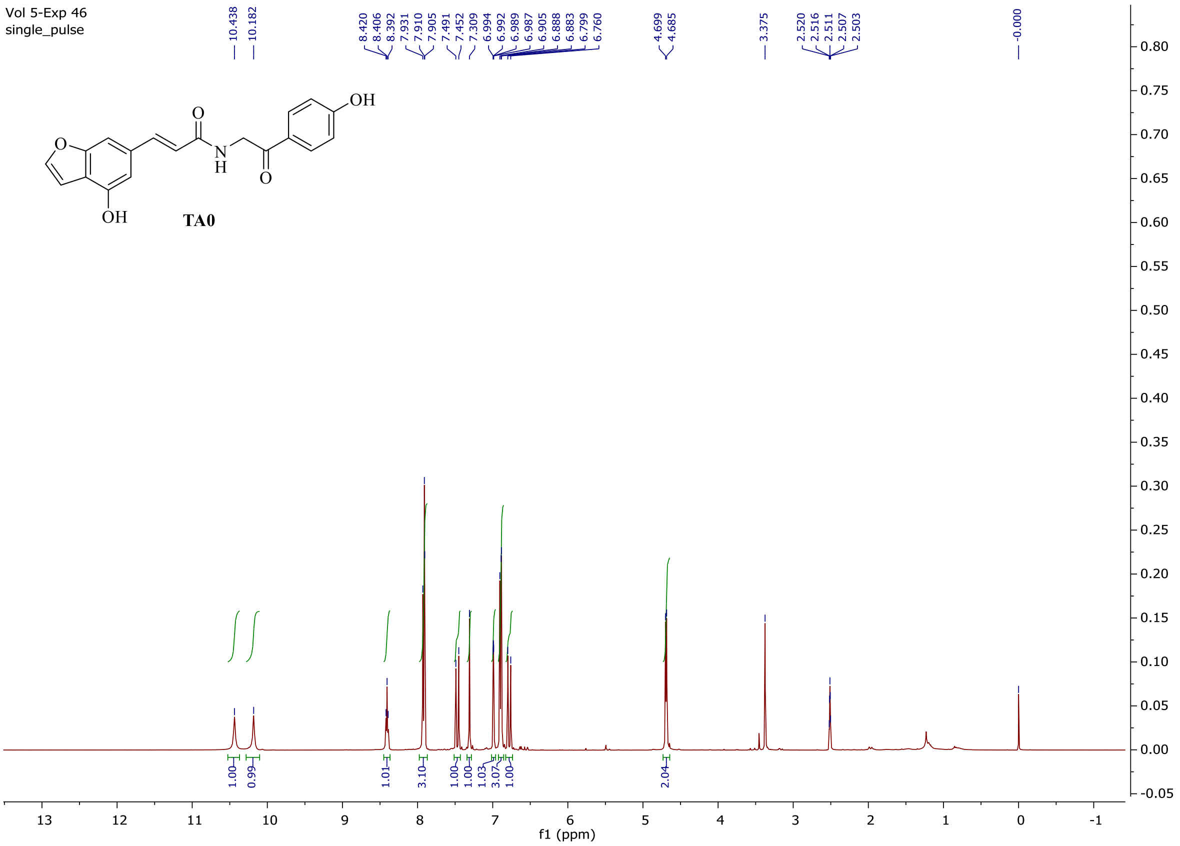
Vol 5-Exp 46
single_pulse
TA0
10.438
10.182
8.420
8.406
8.392
7.931
7.910
7.905
7.491
7.452
7.309
6.994
6.992
6.989
6.987
6.905
6.888
6.883
6.799
6.760
4.699
4.685
3.375
2.520
2.516
2.511
2.507
2.503
-0.000
1.00
0.99
1.01
3.10
1.00
1.00
1.03
3.07
1.00
2.04
f1 (ppm)

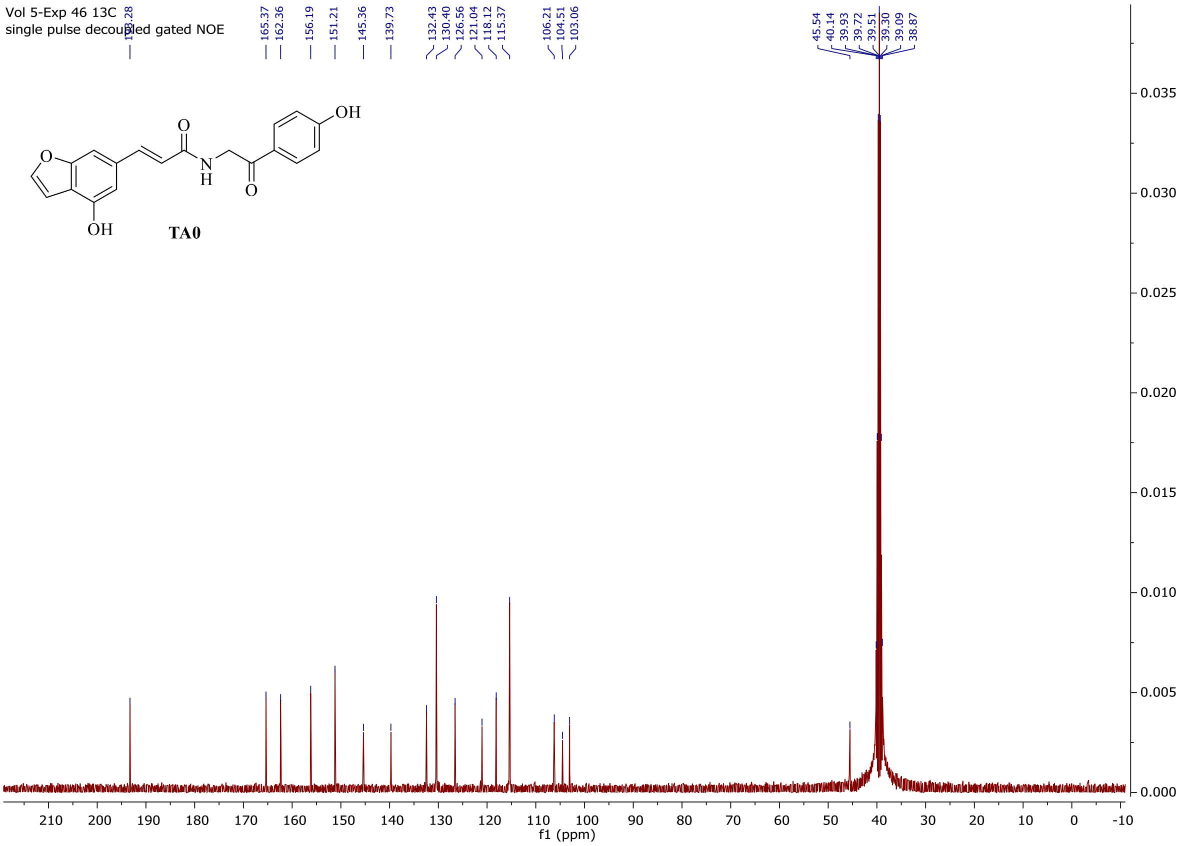
Vol 5-Exp 46 13C
single pulse decoupled gated NOE
TA0
165.37
162.36
156.19
151.21
145.36
139.73
132.43
130.40
126.56
121.04
118.12
115.37
106.21
104.51
103.06
45.54
40.14
39.93
39.72
39.51
39.30
39.09
38.87
f1 (ppm)

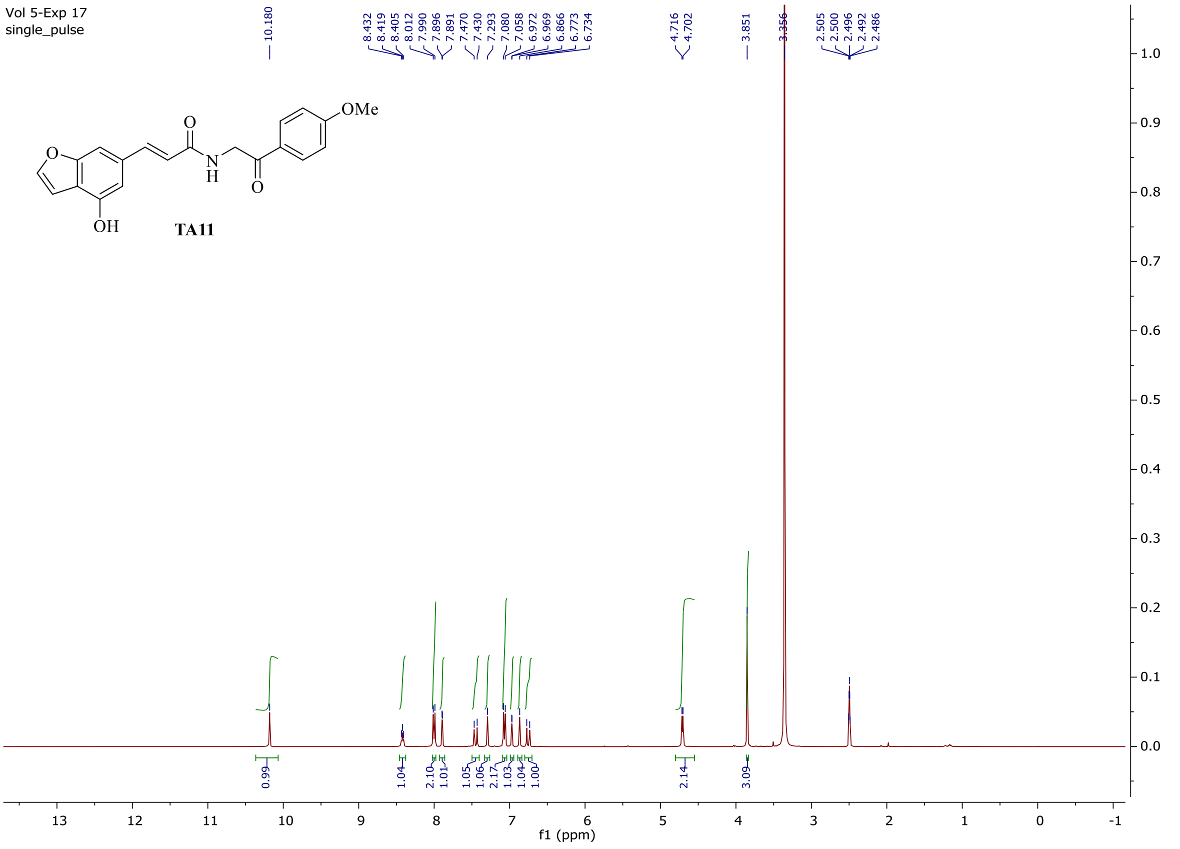
Vol 5-Exp 17
single_pulse
10.180
8.432
8.419
8.405
8.012
7.990
7.896
7.891
7.470
7.430
7.293
7.080
7.058
6.972
6.969
6.866
6.773
6.734
4.716
4.702
3.851
3.356
2.505
2.500
2.496
2.492
2.486
OMe
O
N
H
O
O
OH
TA11
0.99
1.04
2.10
1.01
1.05
1.06
2.17
1.03
1.04
1.00
2.14
3.09
f1 (ppm)

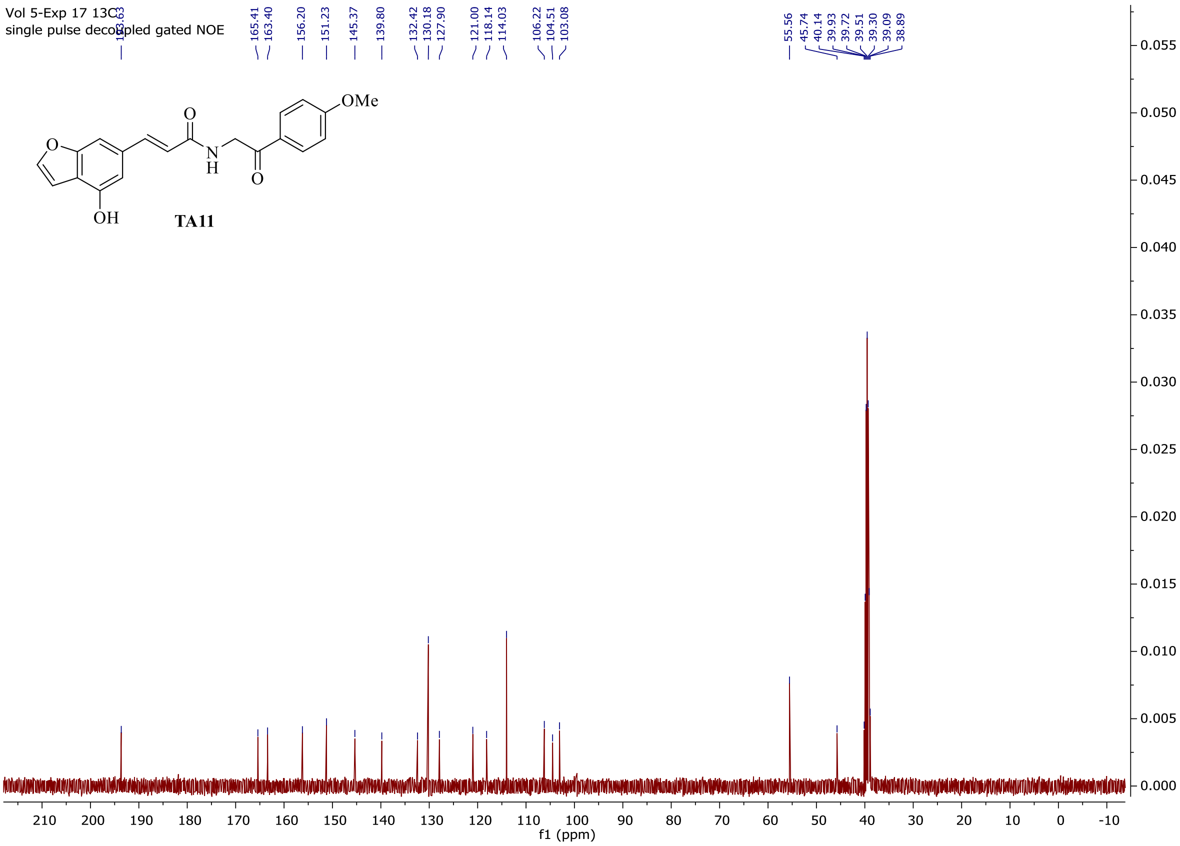
Vol 5-Exp 17 13C
single pulse decoupled gated NOE
165.41
163.40
156.20
151.23
145.37
139.80
132.42
130.18
127.90
121.00
118.14
114.03
106.22
104.51
103.08
55.56
45.74
40.14
39.93
39.72
39.51
39.30
39.09
38.89
OMe
O
N
H
O
O
OH
TA11
f1 (ppm)

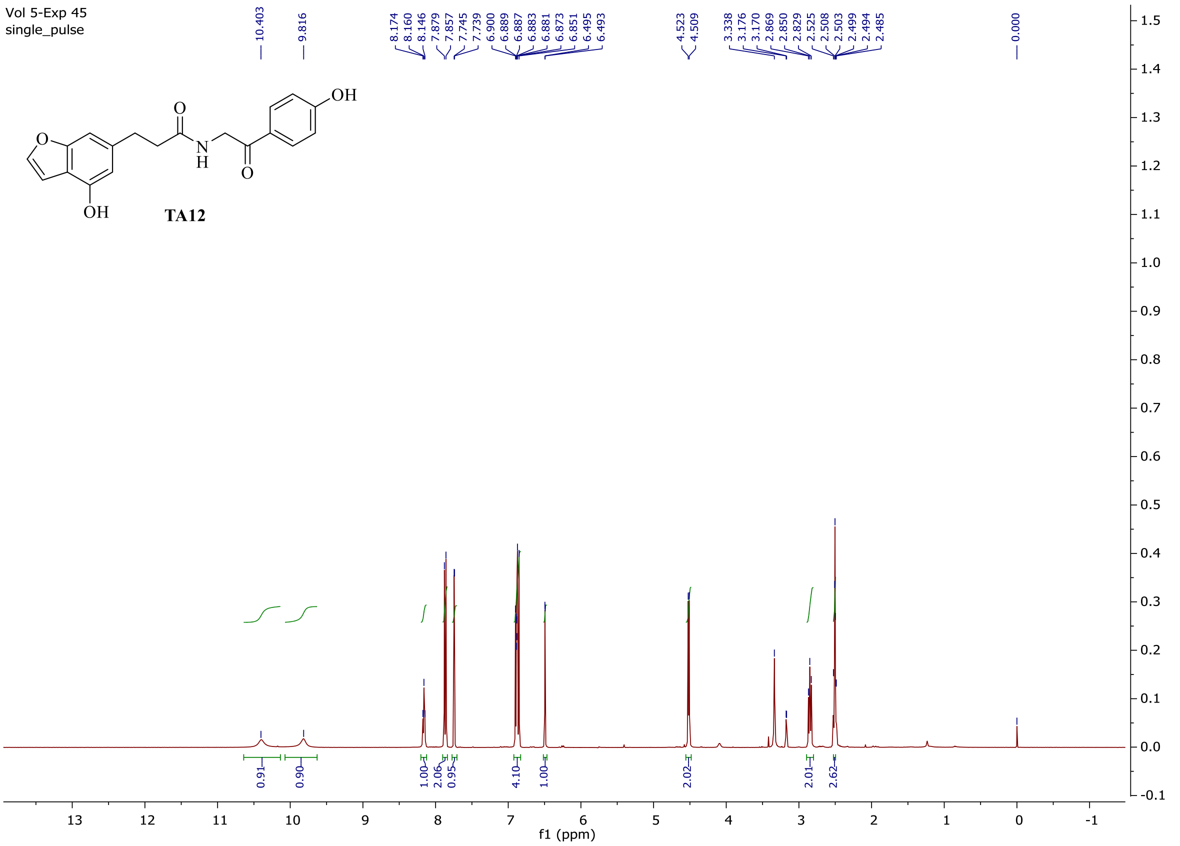
Vol 5-Exp 45
single_pulse
10.403
9.816
8.174
8.160
8.146
7.879
7.857
7.745
7.739
6.900
6.889
6.887
6.883
6.881
6.873
6.851
6.495
6.493
4.523
4.509
3.338
3.176
3.170
2.869
2.850
2.829
2.525
2.508
2.503
2.499
2.494
2.485
0.000
OH
O
O
N
H
O
OH
TA12
0.91
0.90
1.00
2.06
0.95
4.10
1.00
2.02
2.01
2.62
f1 (ppm)

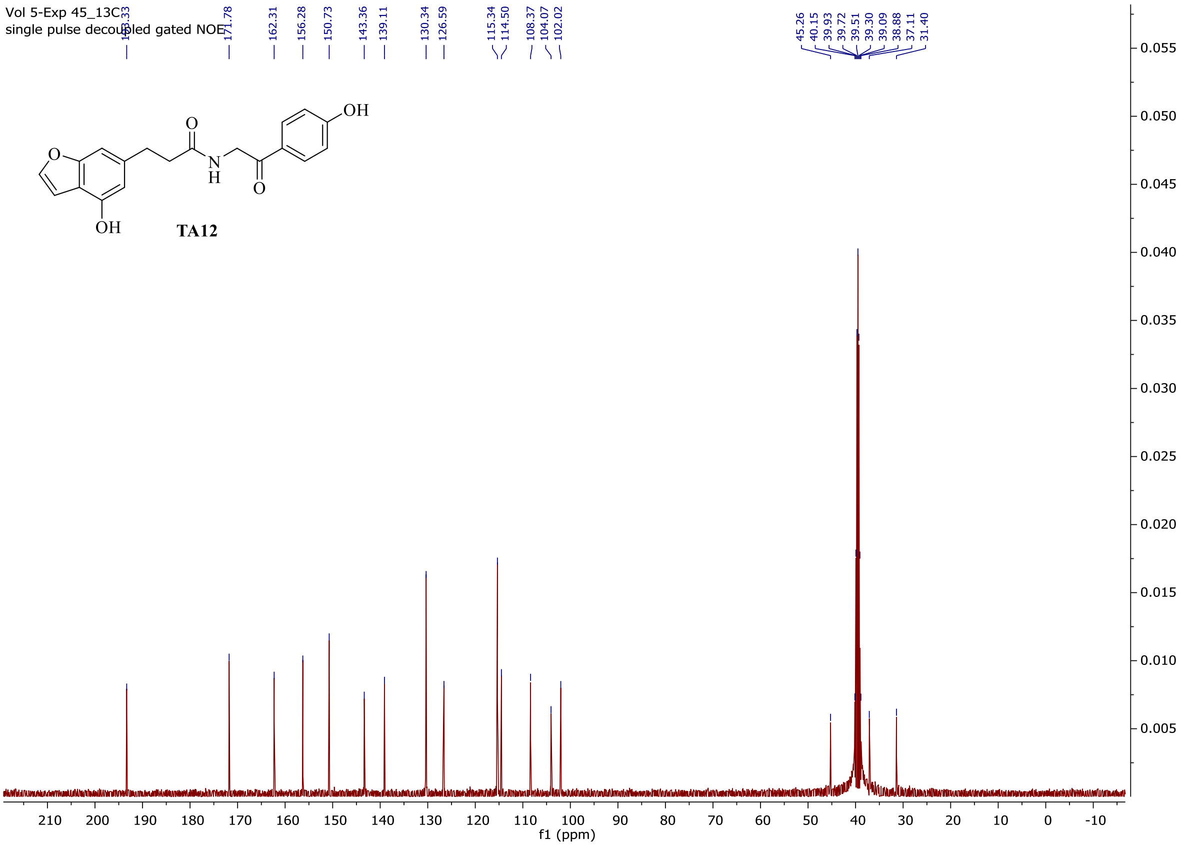
Vol 5-Exp 45_13C
single pulse decoupled gated NOE
193.33
171.78
162.31
156.28
150.73
143.36
139.11
130.34
126.59
115.34
114.50
108.37
104.07
102.02
45.26
40.15
39.93
39.72
39.51
39.30
39.09
38.88
37.11
31.40
OH
O
O
N
H
O
OH
TA12
f1 (ppm)

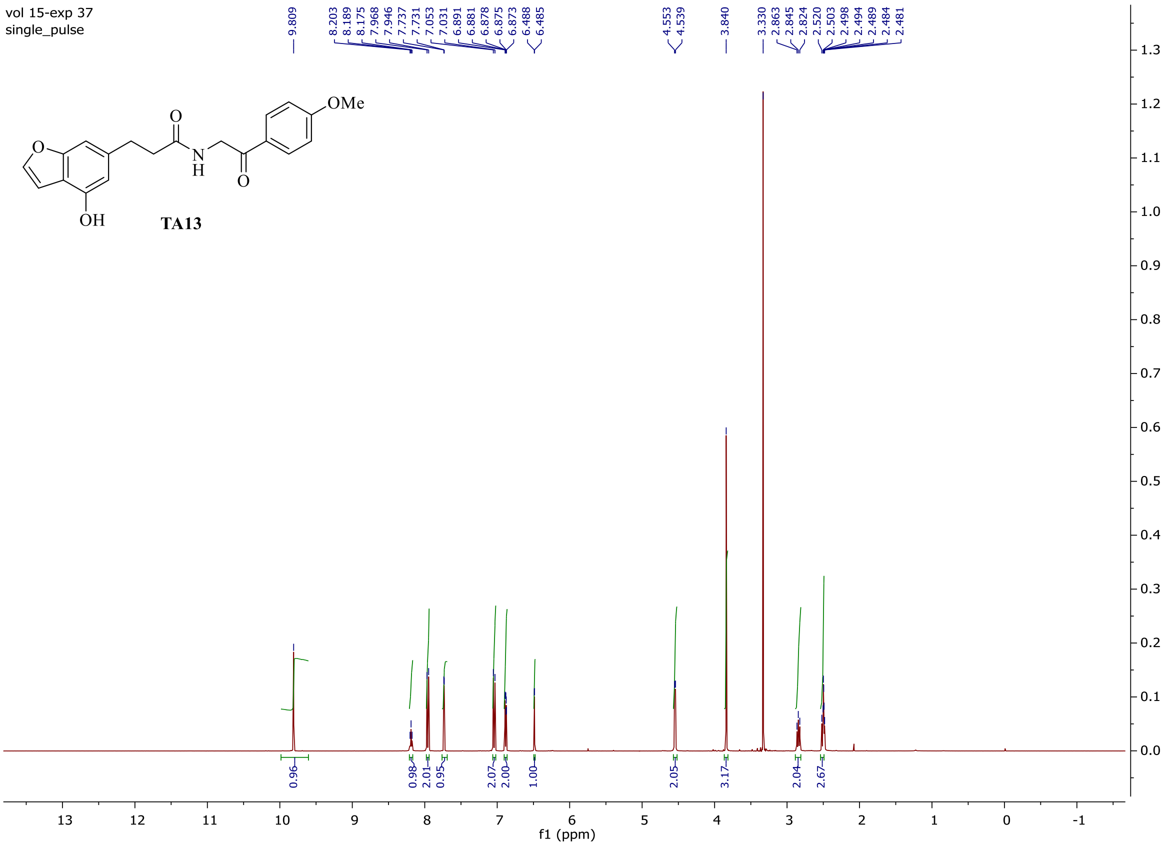
vol 15-exp 37
single_pulse
OMe
O
O
N
H
O
OH
TA13
f1 (ppm)

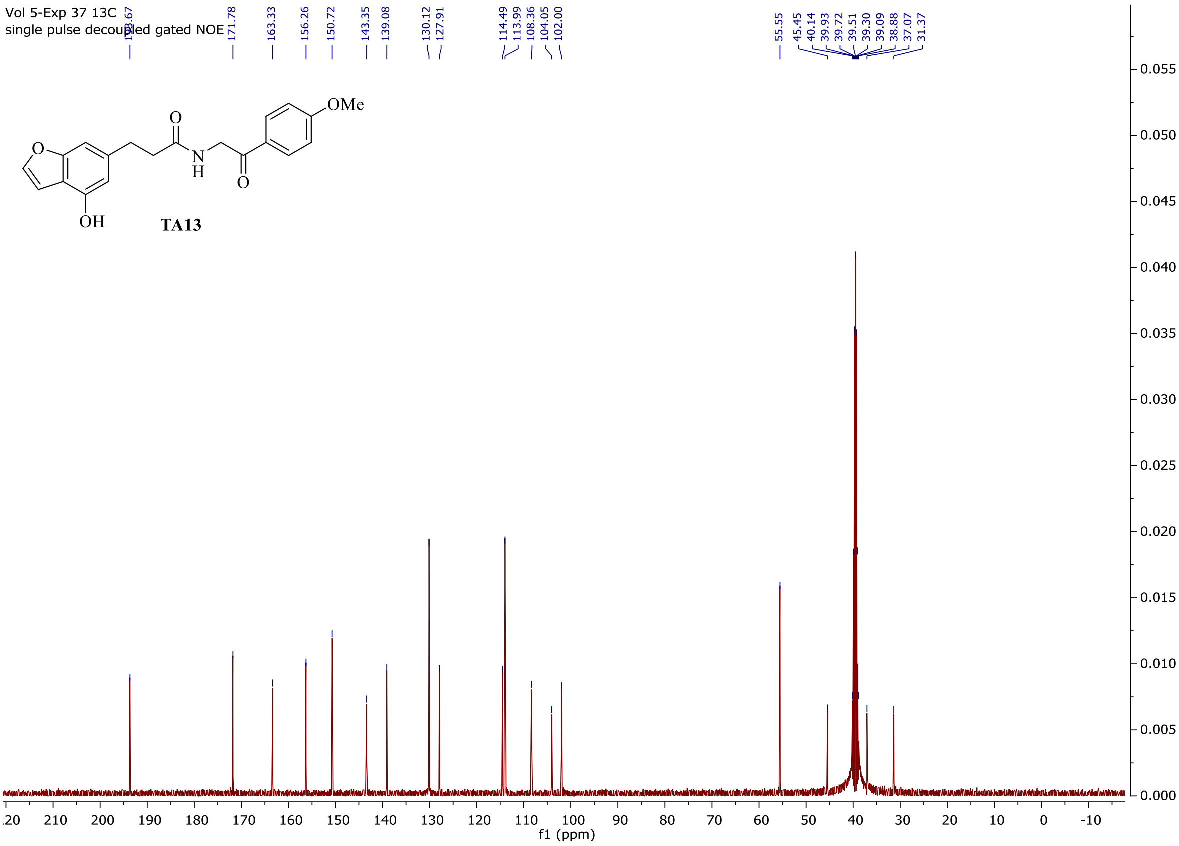
Vol 5-Exp 37 13C
single pulse decoupled gated NOE
OMe
O
O
N
H
O
OH
TA13
f1 (ppm)

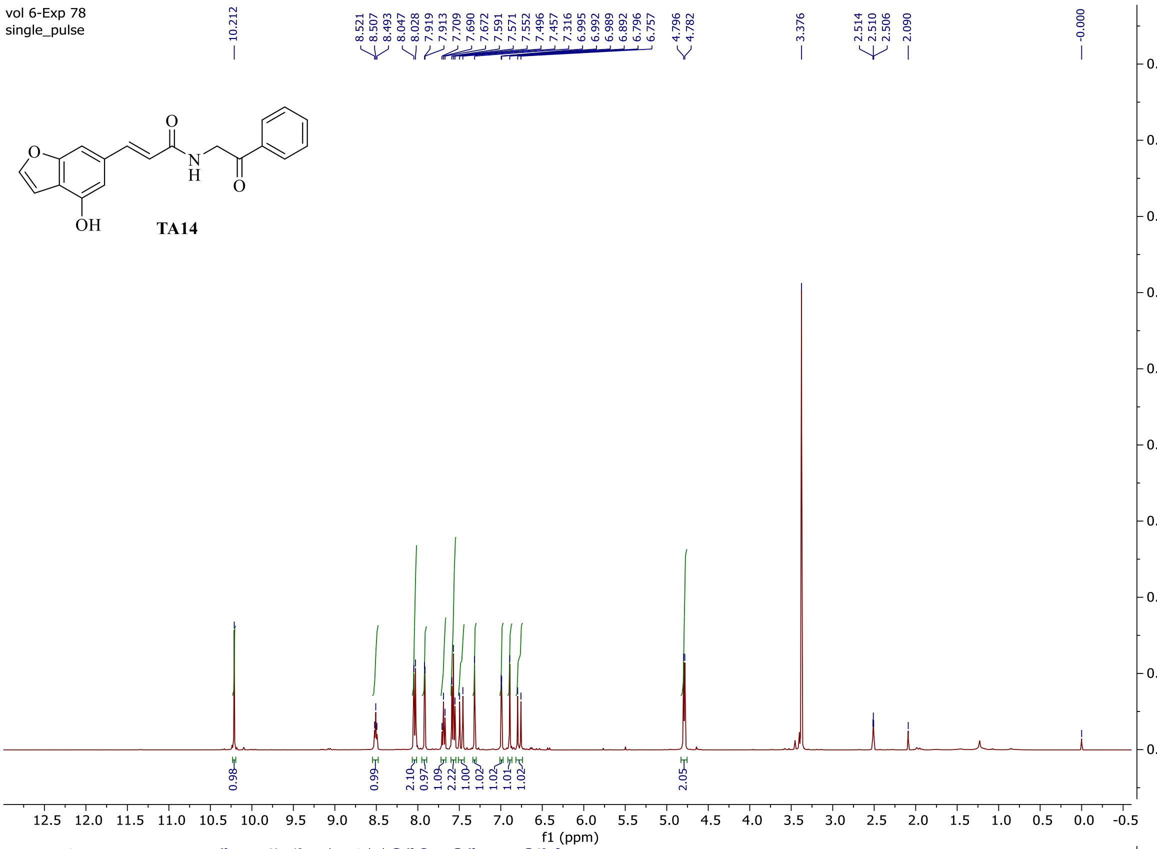
vol 6-Exp 78
single_pulse
10.212
8.521 8.507 8.493 8.047 8.028 7.919 7.913 7.709 7.690 7.672 7.591 7.571 7.552 7.496 7.457 7.316 6.995 6.992 6.989 6.892 6.796 6.757
4.796 4.782
3.376
2.514 2.510 2.506
2.090
-0.000
TA14
0.98 0.99 2.10 0.97 1.09 2.22 1.00 1.02 1.02 1.01 1.02 2.05
f1 (ppm)

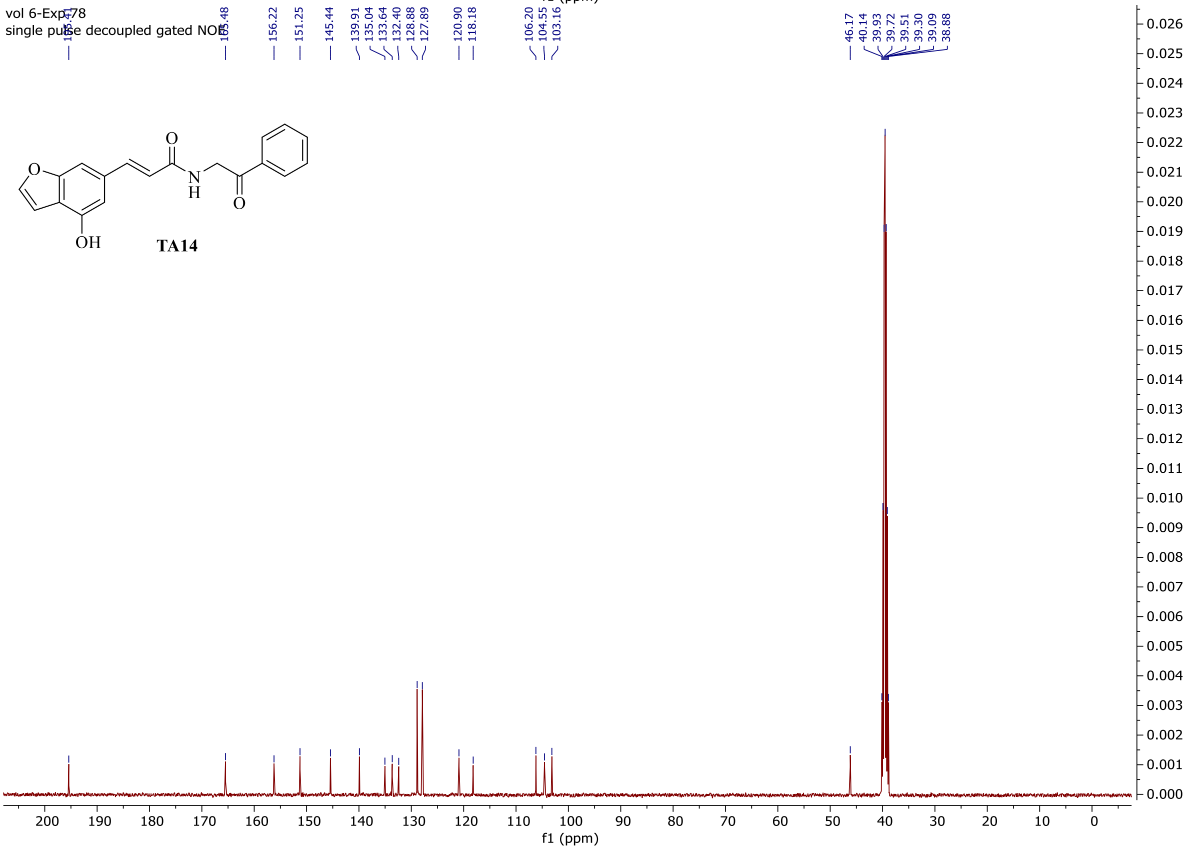
vol 6-Exp 78
single pulse decoupled gated NOE
156.22
151.25
145.44
139.91 135.04 133.64 132.40 128.88 127.89
120.90 118.18
106.20 104.55 103.16
46.17 40.14 39.93 39.72 39.51 39.30 39.09 38.88
TA14
f1 (ppm)

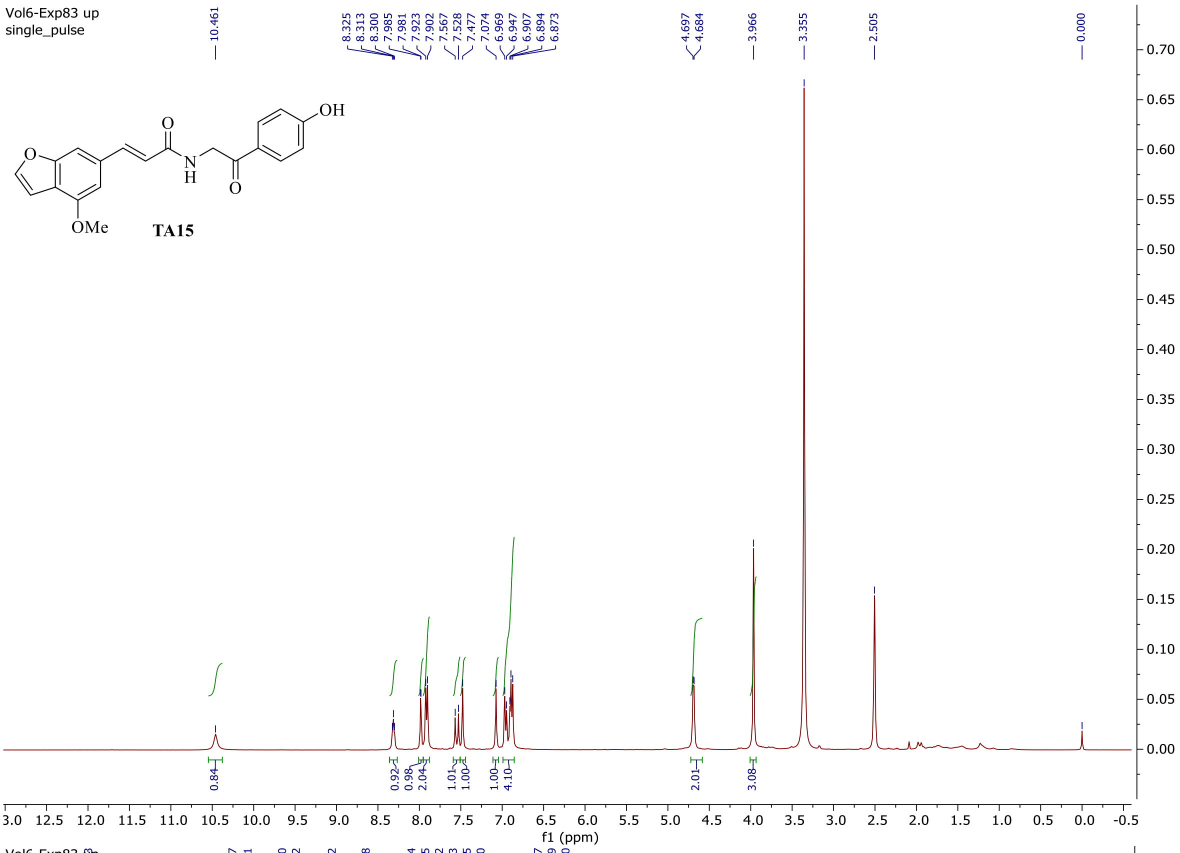
Vol6-Exp83 up
single_pulse
10.461
8.325 8.313 8.300 7.985 7.981 7.923 7.902 7.567 7.528 7.477 7.074 6.969 6.947 6.907 6.894 6.873
4.697 4.684
3.966
3.355
2.505
0.000
0.84
0.92 0.98 2.04
1.01 1.00
1.00 4.10
2.01
3.08
OH
O
O
N
H
O
OMe
TA15
f1 (ppm)

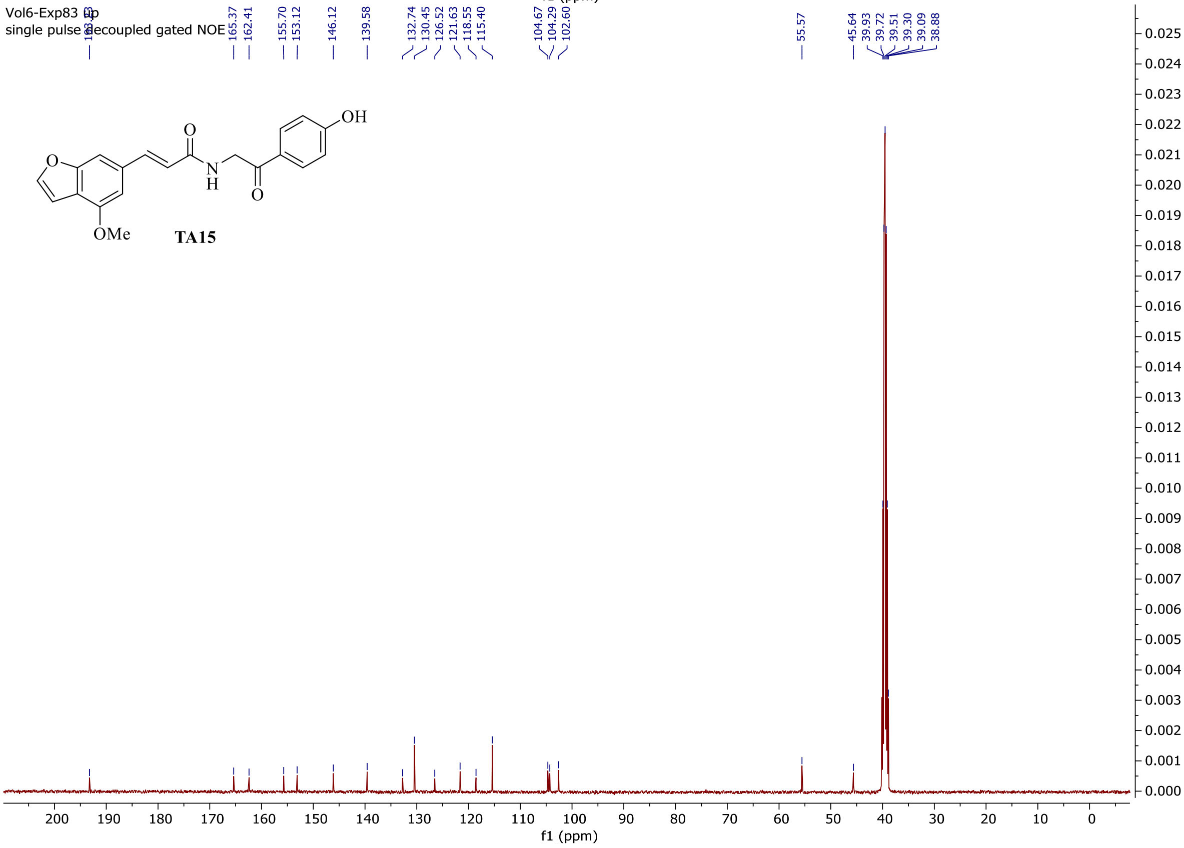
Vol6-Exp83 up
single pulse decoupled gated NOE
165.37
162.41
155.70
153.12
146.12
139.58
132.74 130.45 126.52 121.63 118.55 115.40
104.67 104.29 102.60
55.57
45.64 39.93 39.72 39.51 39.30 39.09 38.88
OH
O
O
N
H
O
OMe
TA15
f1 (ppm)

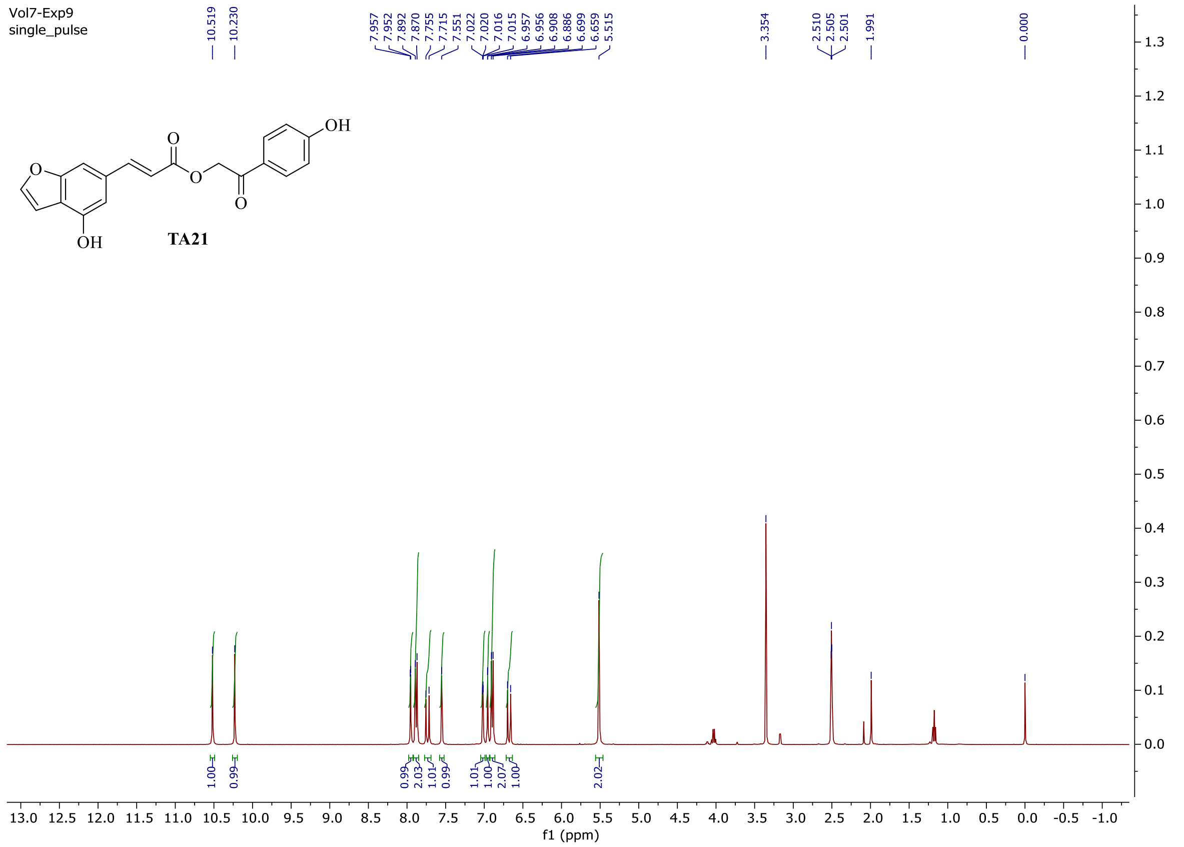
Vol7-Exp9
single_pulse
TA21
f1 (ppm)

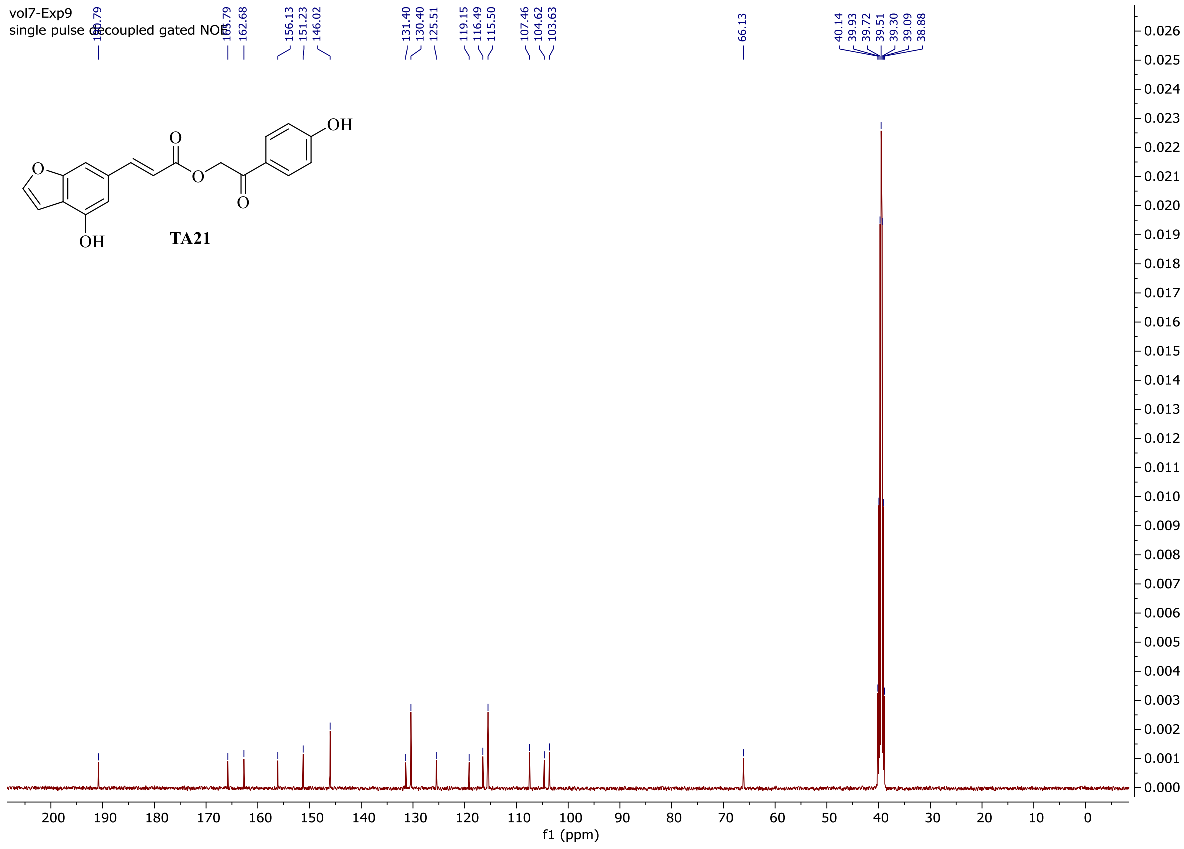
vol7-Exp9
single pulse decoupled gated NOE
TA21
f1 (ppm)

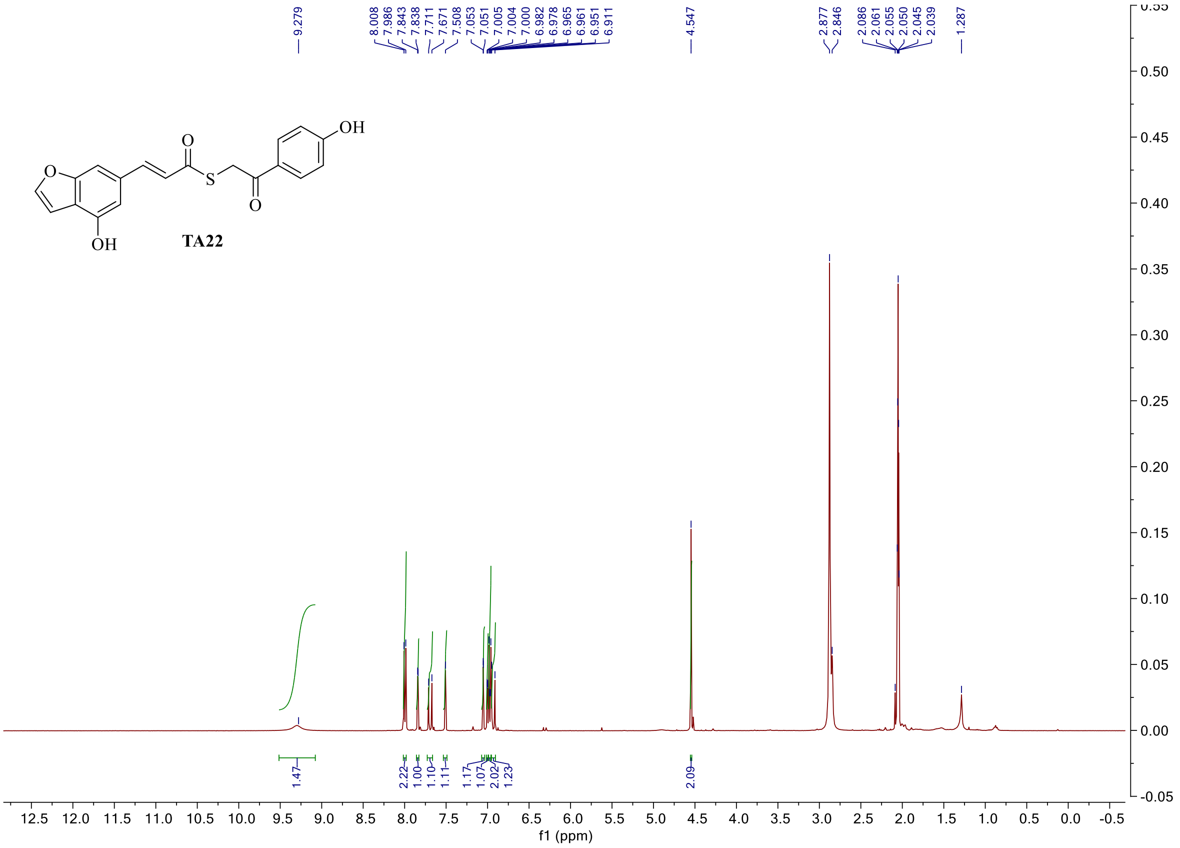

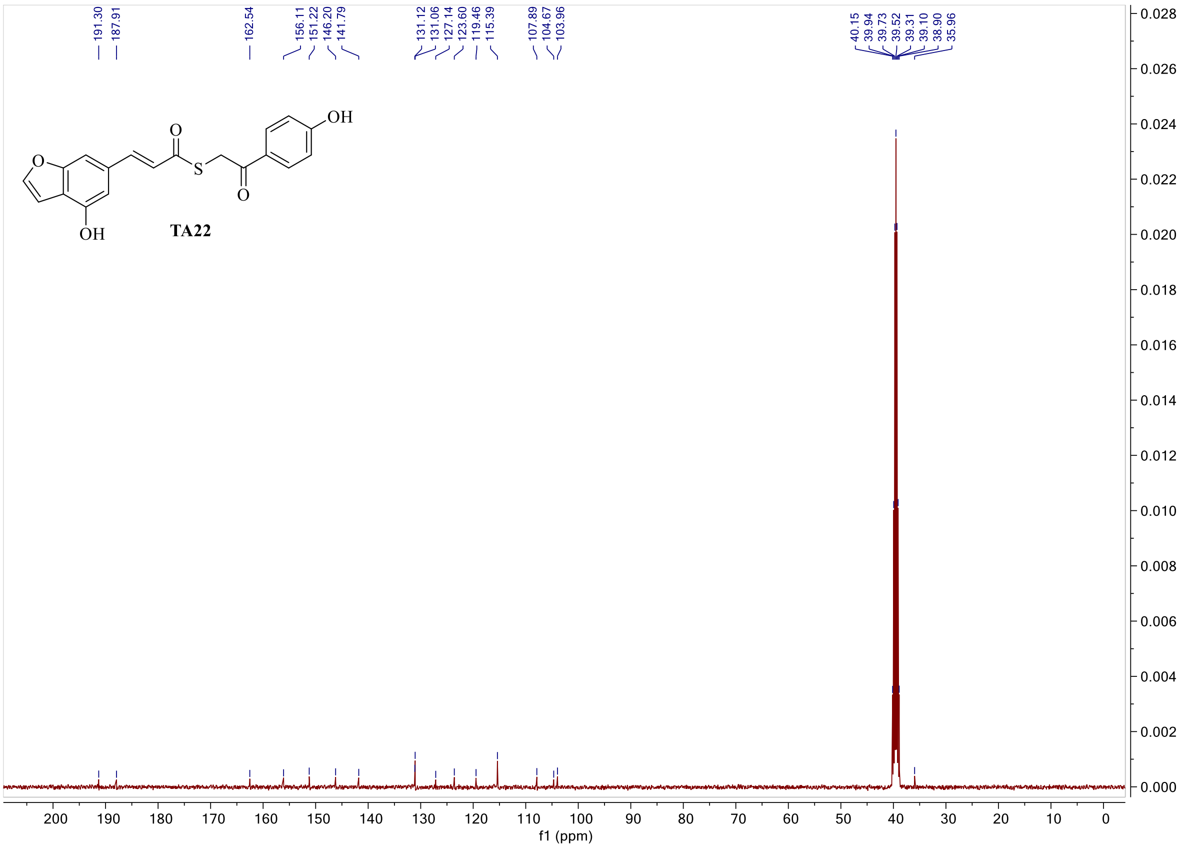

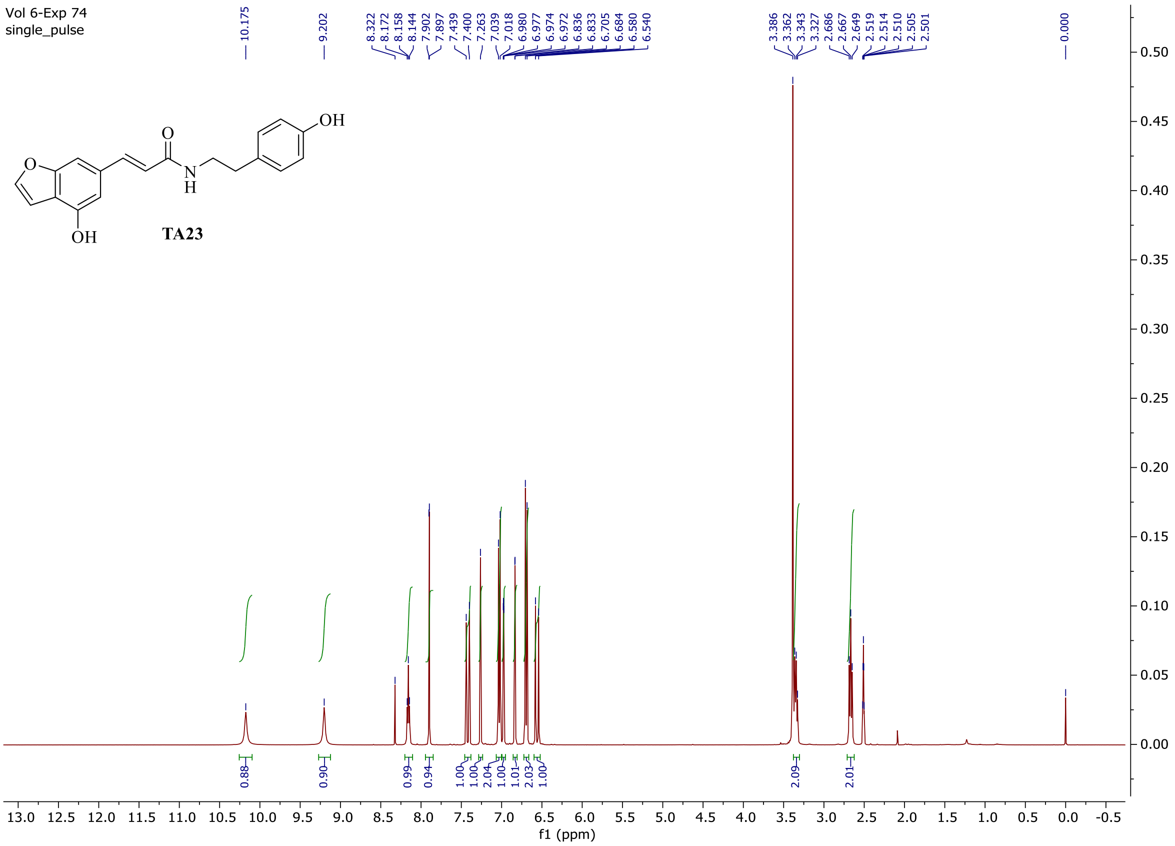
Vol 6-Exp 74
single_pulse
10.175
9.202
8.322
8.172
8.158
8.144
7.902
7.897
7.439
7.400
7.263
7.039
7.018
6.980
6.977
6.974
6.972
6.836
6.833
6.705
6.684
6.580
6.540
3.386
3.362
3.343
3.327
2.686
2.667
2.649
2.519
2.514
2.510
2.505
2.501
0.000
OH
O
O
N
H
OH
TA23
0.88
0.90
0.99
0.94
1.00
1.00
2.04
1.00
1.01
2.03
1.00
2.09
2.01
f1 (ppm)

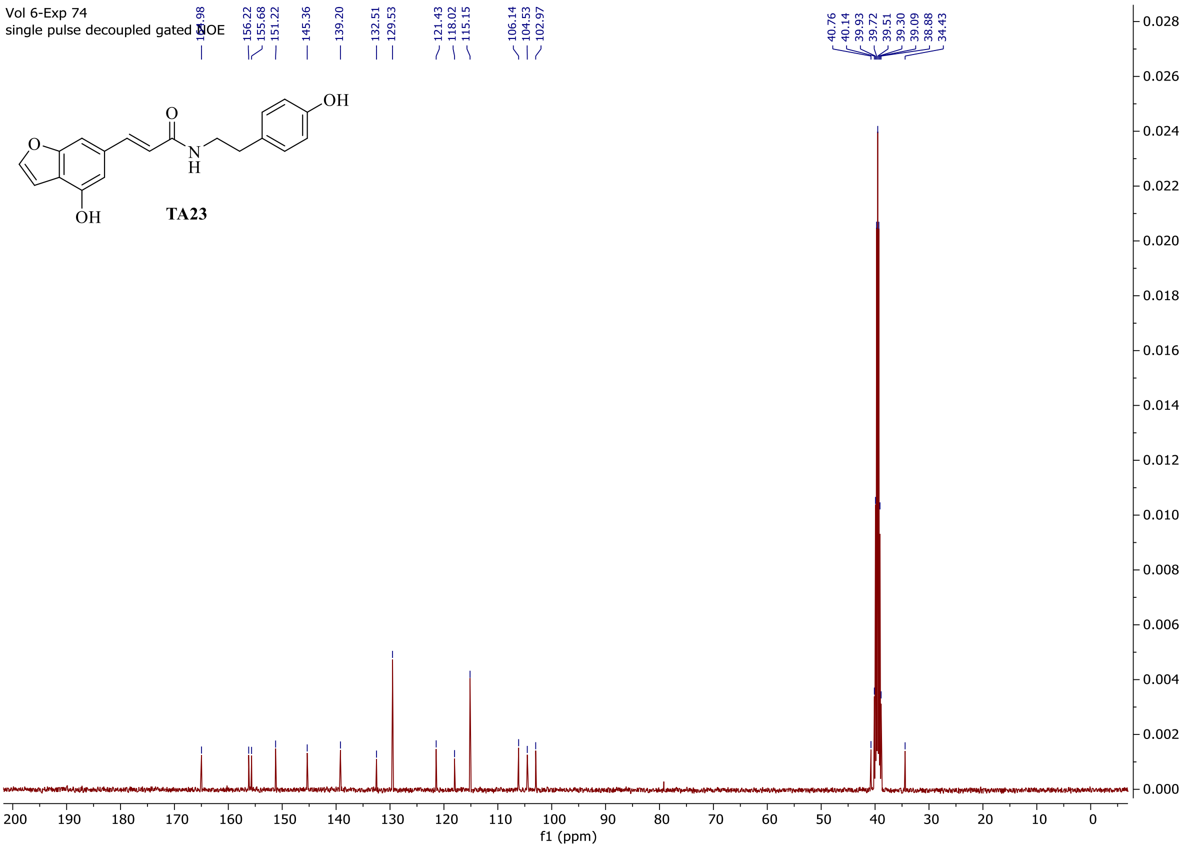
Vol 6-Exp 74
single pulse decoupled gated NOE
156.22
155.68
151.22
145.36
139.20
132.51
129.53
121.43
118.02
115.15
106.14
104.53
102.97
40.76
40.14
39.93
39.72
39.51
39.30
39.09
38.88
34.43
OH
O
O
N
H
OH
TA23
f1 (ppm)

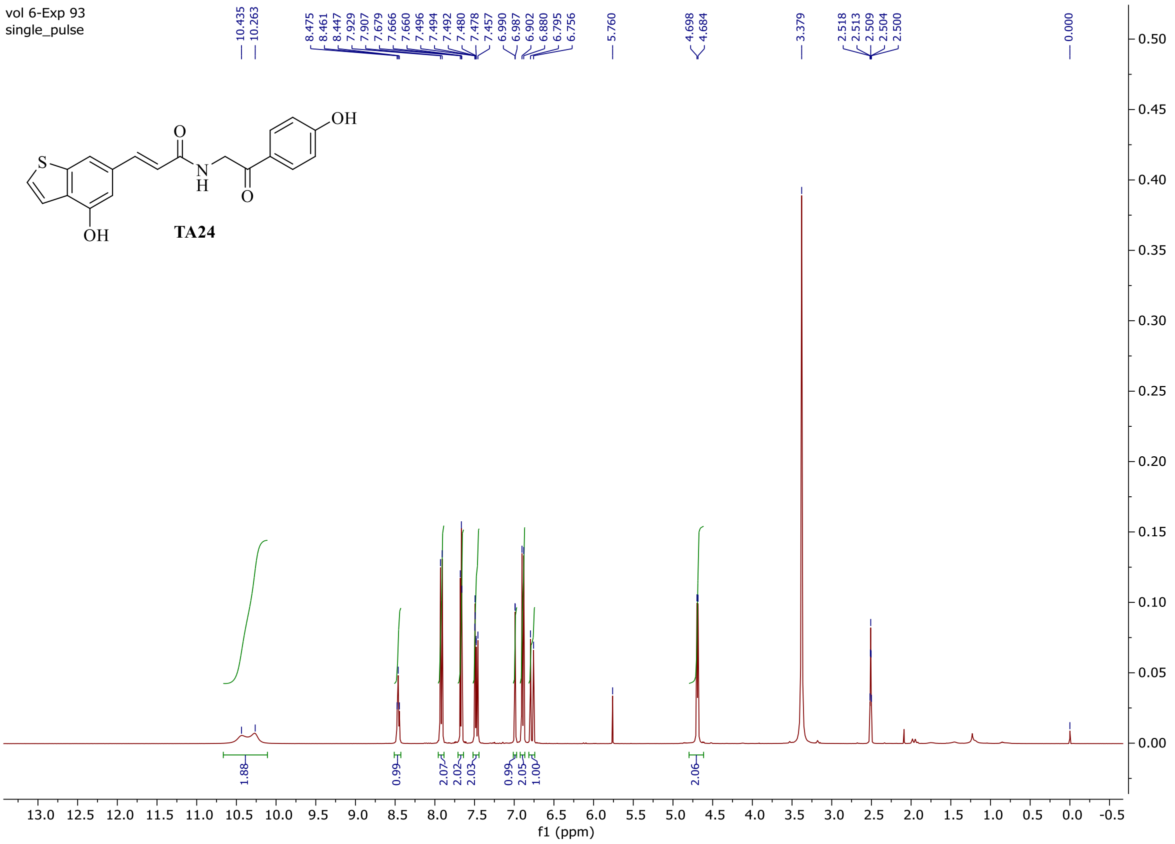

vol 6-Exp 93
single_pulse
10.435
10.263
8.475
8.461
8.447
7.929
7.907
7.679
7.666
7.660
7.496
7.494
7.492
7.480
7.478
7.457
6.990
6.987
6.902
6.880
6.795
6.756
5.760
4.698
4.684
3.379
2.518
2.513
2.509
2.504
2.500
0.000
S
O
N
H
O
OH
OH
TA24
1.88
0.99
2.07
2.02
2.03
0.99
2.05
1.00
2.06
f1 (ppm)

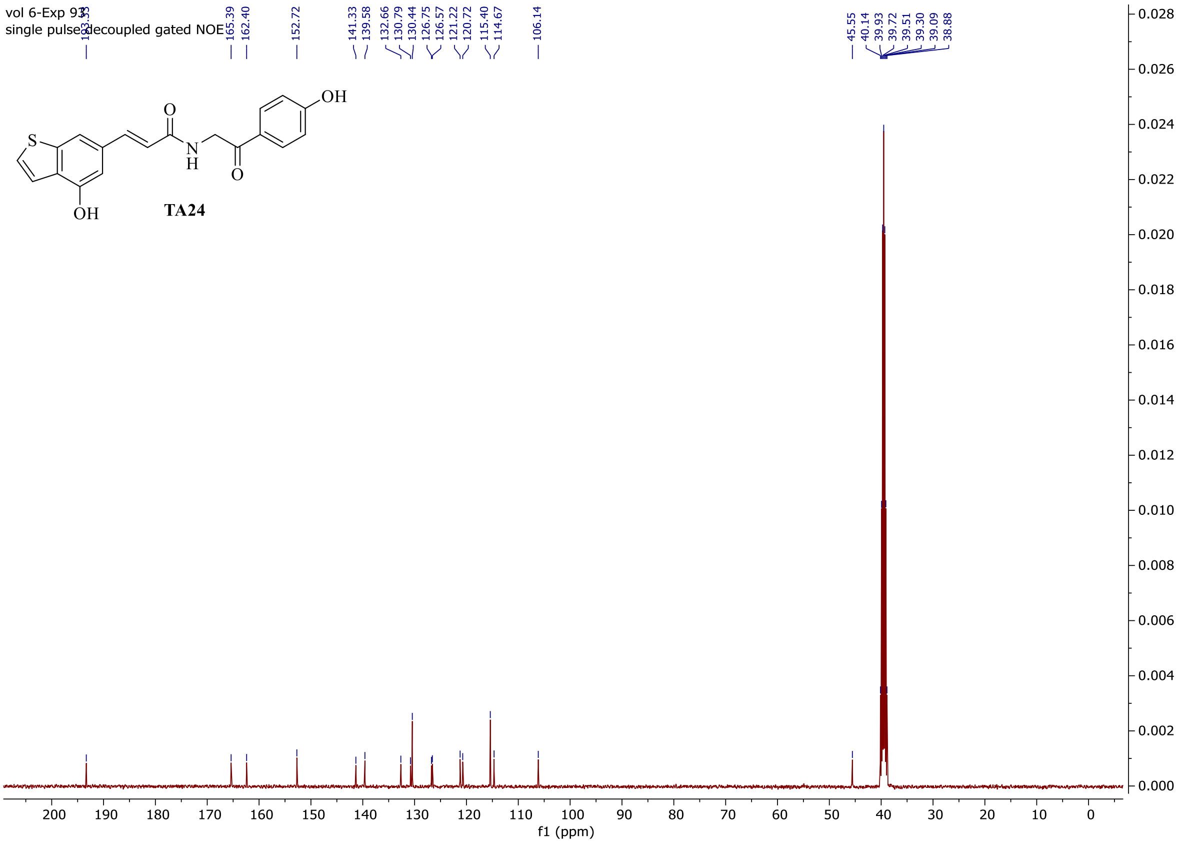

vol 6-Exp 93
single pulse decoupled gated NOE
165.39
162.40
152.72
141.33
139.58
132.66
130.79
130.44
126.75
126.57
121.22
120.72
115.40
114.67
106.14
45.55
40.14
39.93
39.72
39.51
39.30
39.09
38.88
S
O
N
H
O
OH
OH
TA24
f1 (ppm)

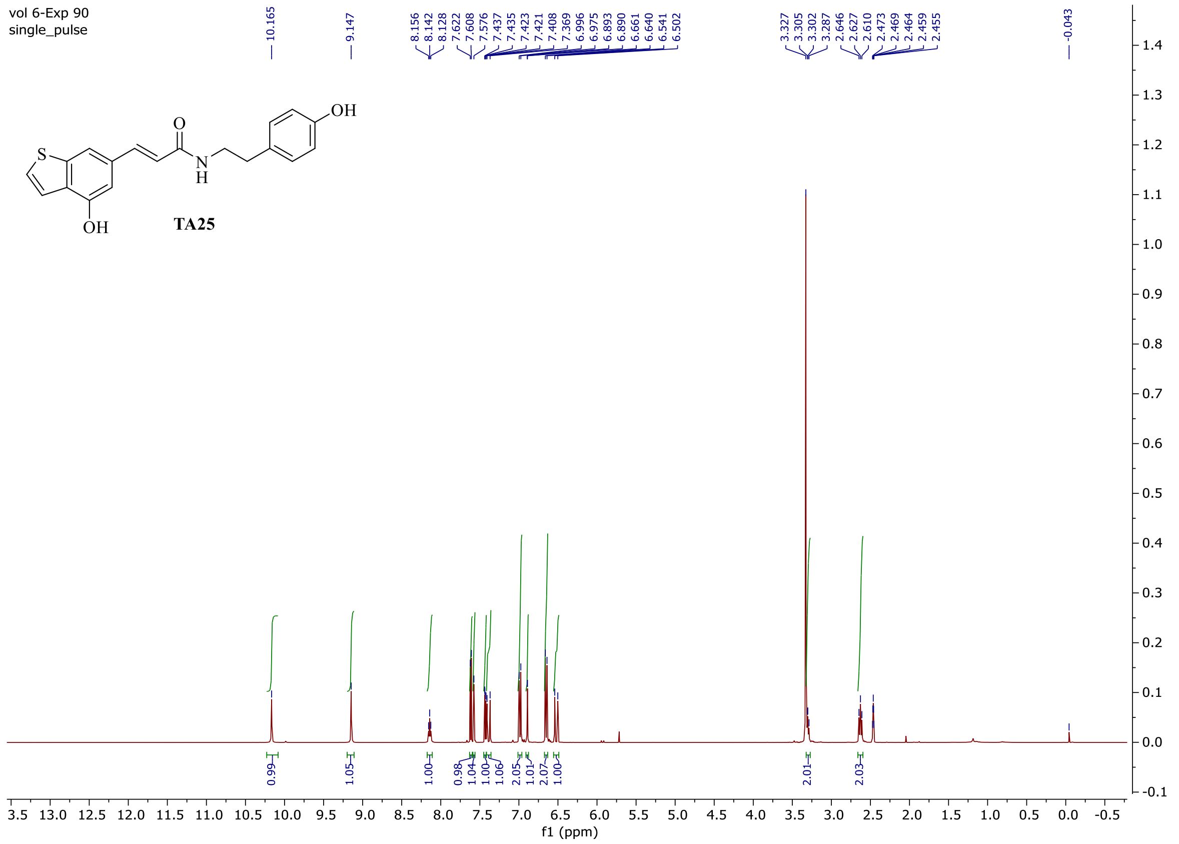
vol 6-Exp 90
single_pulse
10.165
9.147
8.156
8.142
8.128
7.622
7.608
7.576
7.437
7.435
7.423
7.421
7.408
7.369
6.996
6.975
6.893
6.890
6.661
6.640
6.541
6.502
3.327
3.305
3.302
3.287
2.646
2.627
2.610
2.473
2.469
2.464
2.459
2.455
-0.043
OH
O
S
N
H
OH
TA25
0.99
1.05
1.00
0.98
1.04
1.00
1.06
2.05
1.01
2.07
1.00
2.01
2.03
f1 (ppm)

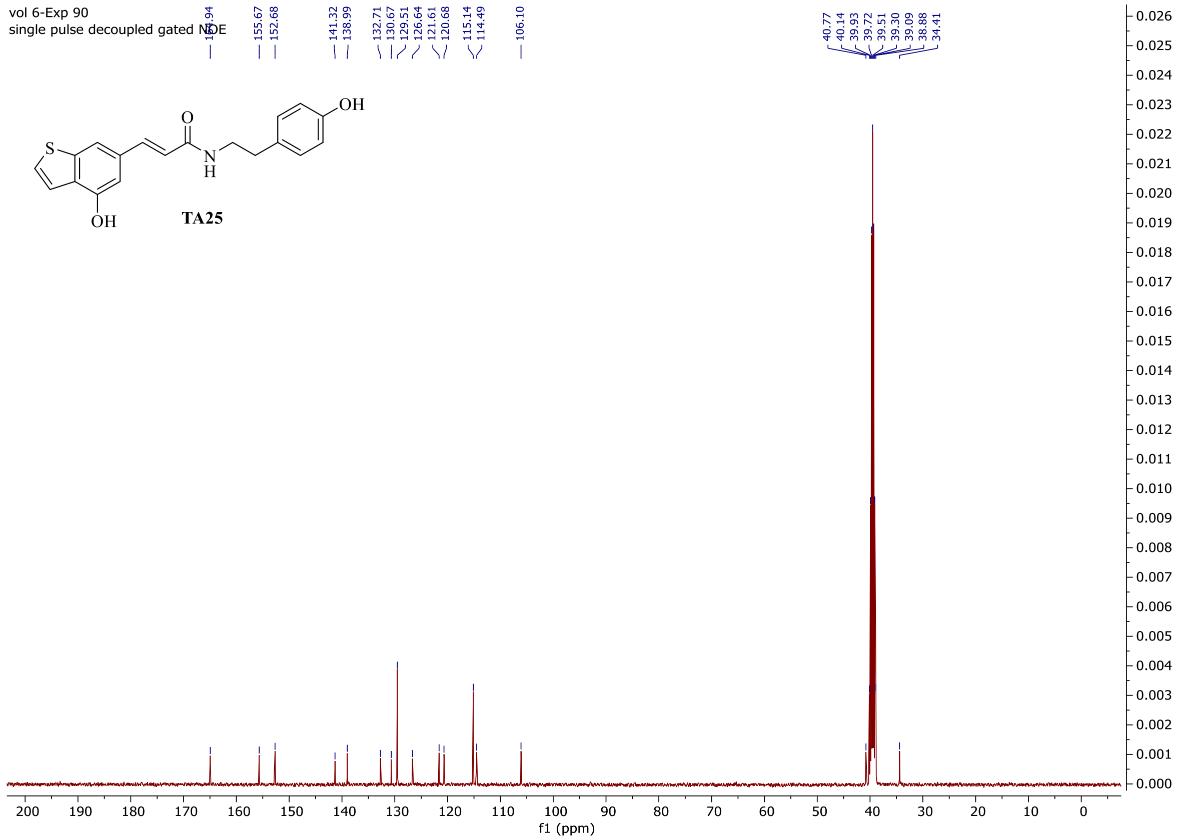
vol 6-Exp 90
single pulse decoupled gated NOE
155.67
152.68
141.32
138.99
132.71
130.67
129.51
126.64
121.61
120.68
115.14
114.49
106.10
40.77
40.14
39.93
39.72
39.51
39.30
39.09
38.88
34.41
OH
O
S
N
H
OH
TA25
f1 (ppm)
